# Targeting PTPRZ inhibits stem cell-like properties and tumorigenicity in glioblastoma cells

**DOI:** 10.1038/s41598-017-05931-8

**Published:** 2017-07-17

**Authors:** Akihiro Fujikawa, Hajime Sugawara, Taisaku Tanaka, Masahito Matsumoto, Kazuya Kuboyama, Ryoko Suzuki, Naomi Tanga, Atsuto Ogata, Makoto Masumura, Masaharu Noda

**Affiliations:** 1 Division of Molecular Neurobiology, National Institute for Basic Biology (NIBB), 5-1 Higashiyama, Myodaiji-cho, Okazaki, Aichi 444-8787 Japan; 2 Asubio Pharma Co., Ltd., 6-4-3 Minatojima-Minamimachi, Chuo-ku, Kobe, Hyogo, 650-0047 Japan; 3 School of Life Science, The Graduate University for Advanced Studies (SOKENDAI), 5-1 Higashiyama, Myodaiji-cho, Okazaki, Aichi 444-8787 Japan

## Abstract

The R5 subfamily of receptor-type protein tyrosine phosphatases (RPTPs) comprises PTPRZ and PTPRG. A recent study on primary human glioblastomas suggested a close association between *PTPRZ1* (human *PTPRZ*) expression and cancer stemness. However, the functional roles of PTPRZ activity in glioma stem cells have remained unclear. In the present study, we found that sphere-forming cells from the rat C6 and human U251 glioblastoma cell lines showed high expression levels of PTPRZ-B, the short receptor isoform of PTPRZ. Stable *PTPRZ* knockdown altered the expression levels of stem cell transcription factors such as SOX2, OLIG2, and POU3F2 and decreased the sphere-forming abilities of these cells. Suppressive effects on the cancer stem-like properties of the cells were also observed following the knockdown of *PTPRG*. Here, we identified NAZ2329, a cell-permeable small molecule that allosterically inhibits both PTPRZ and PTPRG. NAZ2329 reduced the expression of SOX2 in C6 and U251 cells and abrogated the sphere-forming abilities of these cells. Tumor growth in the C6 xenograft mouse model was significantly slower with the co-treatment of NAZ2329 with temozolomide, an alkylating agent, than with the individual treatments. These results indicate that pharmacological inhibition of R5 RPTPs is a promising strategy for the treatment of malignant gliomas.

## Introduction

Glioblastoma has been classified by the WHO as the highest grade glioma (grade IV). Malignant glioma therapy currently involves surgical resection followed by adjuvant chemoradiotherapy. However, the median survival rate of patients with glioblastoma is 14 months^1^. The lack of effective therapeutic options indicates an unmet medical need for patients with glioblastoma. Protein tyrosine phosphorylation controls many cellular functions, and its dysregulation has been implicated in the etiology of various human cancers, including gliomas^2, 3^. In contrast to oncogenic protein tyrosine kinases (PTKs), protein tyrosine phosphatases (PTPs) have generally been assumed to act as tumor suppressors.

PTPs have long been recognized as “undruggable” targets, despite their importance in regulating cellular processes and diseases, including cancers^4^. This view is mostly attributable to their highly conserved and positively charged active-site pockets. Many PTP inhibitors have been developed in the past two decades. However, competitive inhibitors that target the active-site Cys residue have phosphotyrosine-mimetic moieties, and their negatively charged groups, which include sulfates, hamper cell permeability^5^. This is also the case for our first small molecule inhibitor of PTPRZ, SCB4380, which is cell impermeable due to the presence of three sulfonic acid groups^6^. However, the recent discovery of allosteric inhibitors of PTP1B, such as Trodusquemine (refs 7 and 8) and the orally bioavailable SHP2 inhibitor SHP099 (ref. 9), has changed this pessimistic view.

PTPRZ and PTPRG, which structurally resemble one another, are members of the R5 receptor-type tyrosine phosphatase (RPTP) subfamily. Both molecules contain an extracellular carbonic anhydrase (CAH)-like domain and a fibronectin type III-like domain, and two intracellular tyrosine phosphatase domains^10^. The membrane proximal phosphatase domain (D1) is active, but the distal D2 domain is inactive. Three isoforms are generated by alternative splicing from a single *PTPRZ* gene: two transmembrane isoforms, PTPRZ-A and PTPRZ-B, and one secretory isoform, PTPRZ-S (also known as phosphacan); all are preferentially expressed in the central nervous system (CNS) as chondroitin sulfate (CS) proteoglycans^11–13^. In normal animals, PTPRZ receptor isoforms play important roles in maintaining oligodendrocyte precursor cells in an undifferentiated state^14, 15^, and the combination of PTPRZ-A and its extracellular ligand pleiotrophin controls the timing of the differentiation of oligodendrocyte precursor cells *in vivo* (ref. 16). PTPRG has four splicing isoforms: three transmembrane isoforms, PTPRG-A, B, and C, and one secretory isoform, PTPRG-S (ref. 17), which are expressed in many tissues including the brain^18^. The PTPRG isoforms are not proteoglycans^18^.

Despite the significant expression of PTPRG in most high-grade astrocytomas^19^, its pathophysiological importance has remained unclear. PTPRZ (the human ortholog is referred to as PTPRZ1) is strongly expressed in malignant gliomas^20, 21^. The inhibition of PTPRZ attenuates the malignant properties of glioblastoma cells, including cell proliferation and migration *in vitro* and tumor formation *in vivo* ^6, 22, 23^, suggesting that the inhibition of PTPRZ is a potential strategy for the treatment of malignant gliomas. SCB4380 is the first small-molecule inhibitor to target the intracellular PTP domain of PTPRZ (ref. 6). Intracellular delivery of SCB4380 via liposome carriers has previously been found to suppress the robust migratory, proliferative, and growth behaviors of rat C6 glioblastoma cells^6^, thereby underscoring the idea that the inhibition of PTPRZ with small molecules is feasible.

Cancer stem-like cells (CSCs) have been shown to persist in tumors as a distinct population and play unique roles in therapeutic resistance and tumor recurrence. Therefore, therapeutic molecular targets in CSCs are the focus of increasing attention toward improving malignant glioma therapy^24^. In glioblastoma, a core set of transcription factors including SOX2, oligodendrocyte transcription factor 2 (OLIG2), POU class 3 homeobox 2 (POU3F2), and spalt-like transcription factor 2 (SALL2) have been shown to be required for reprogramming differentiated glioblastoma cells into stem-like states^25^. More recently, Patel AP. *et al*. reported that *PTPRZ1* transcripts are strongly expressed in individual cells based on single-cell RNA sequencing of primary human glioblastomas. Analyses of intratumoral heterogeneity revealed that the expression levels of *PTPRZ1* transcripts are markedly varied among individual cells and that the strong expression of these transcripts is closely associated with cancer stemness^26^. PTPRZ1 was thus identified as a stemness classifier gene. However, the role of PTPRZ activity in the maintenance of glioma stem-like cells has not been clarified.

Rat C6 and human U251 glioblastoma cells are widely used as experimental models for studying glioblastoma^6, 27, 28^. We previously showed that the knockdown of *Ptprz* in C6 cells weakens their proliferation and migration abilities^6^. In the present study, we examined whether the R5 RPTP subfamily members PTPRZ and PTPRG are associated with glioma stemness and tumorigenicity in rat C6 and human U251 glioblastoma cells using gene silencing. Furthermore, we developed a cell-permeable small-molecule inhibitor for R5 RPTPs and evaluated the effects of pharmacological inhibition of R5 RPTPs on the stemness and tumorigenicity of glioblastoma cells.

## Results

### Roles of PTPRZ in maintaining the stem cell-like features and tumorigenicity of glioblastoma cells

**Figure 1:**
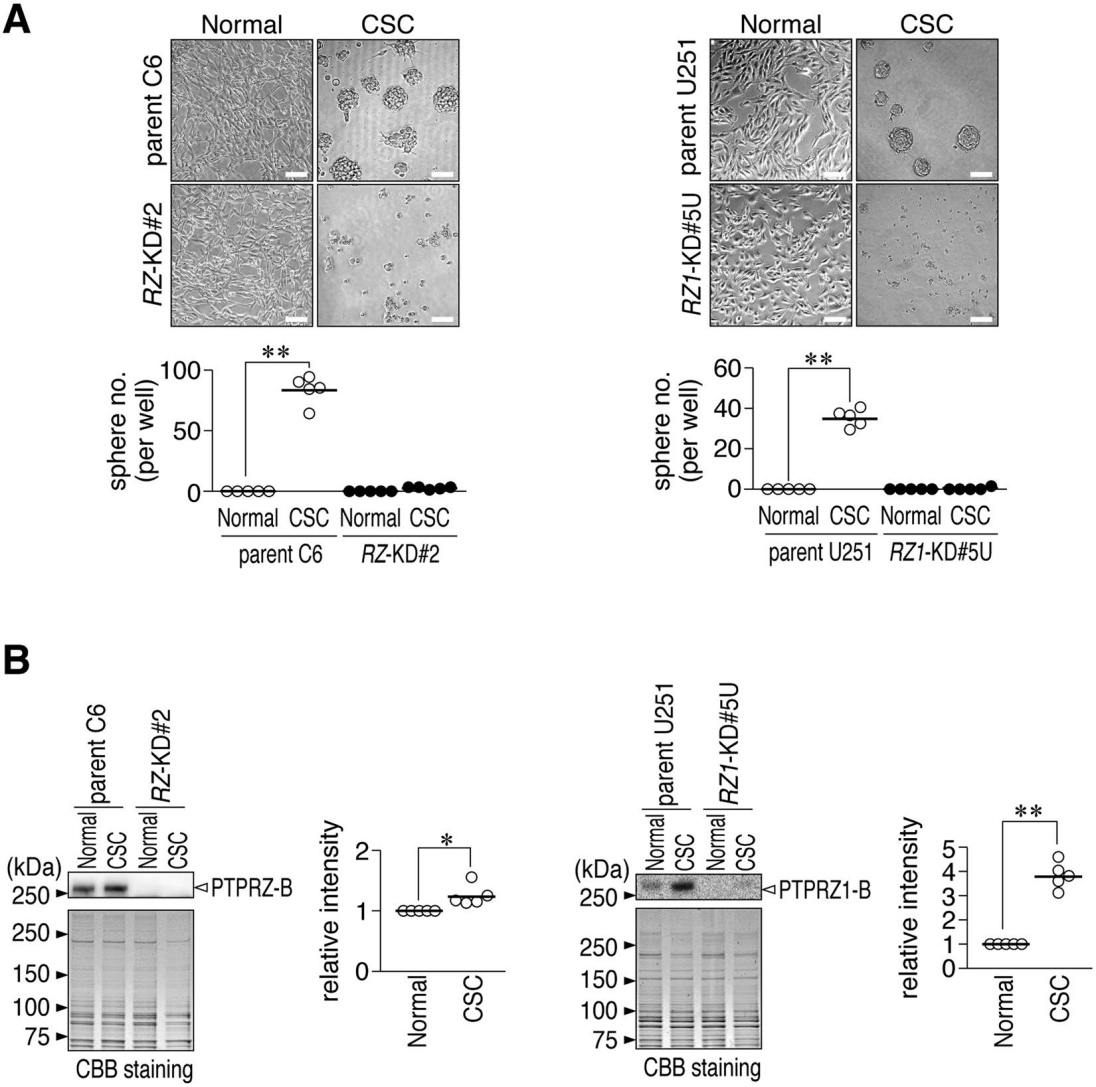
Decreased cell sphere formation of stem cells in *Ptprz*-knockdown glioblastoma cells. (**A**) Sphere formation assay. C6 and U251 cells (parent), *Ptprz-*knockdown C6 cells (*RZ*-KD#2), and *PTPRZ1*-knockdown U251 cells (*RZ1*-KD#5U) were cultured in serum-free cancer stem cell medium for 7 days (CSC) or DMEM supplemented with 10% FBS for 3 days (Normal). Scale bars, 100 µm. Images are representative of five independent cultures. The plot shows the number of cell spheres as the mean value. **P < 0.01 (Student’s *t*-test). (**B**) PTPRZ expression. Cells cultured as in *A* were analyzed by Western blotting with anti-PTPRZ-S (for the detection of rat PTPRZ) and anti-RPTPβ (for human PTPRZ1). The blot is representative of five independent cultures. The plot shows the arbitrary densitometric units of the staining intensity of PTPRZ-B relative to the parental cells cultured in normal serum-containing medium. *P < 0.05, **P < 0.01 (Student’s *t*-test). Full-length blots and gels are presented in Supplementary Fig. S6.

Parental C6 and U251 glioblastoma cells readily formed spheres in serum-free medium supplemented with EGF and FGF (sphere culture conditions) as previously described^29^, whereas *RZ*-KD#2 (a stable *Ptprz*-knockdown clone of C6) and *RZ1*-KD#5U cells (a stable *PTPRZ1*-knockdown clone of U251) did not (Fig. 1A, CSC). Neither cell line formed spheres in serum-supplemented normal medium (Fig. 1A, normal). As suggested by the mRNA expression profiling of human glioblastomas^26^, the protein levels of PTPRZ-B, the major PTPRZ isoform in both glioblastoma cell lines^6, 13^, were 1.2-fold higher in C6 cells and 3.8-fold higher in U251 cells under sphere culture than under normal culture conditions (Fig. 1B). PTPRZ expression was undetectable in the two knockdown clones under both culture conditions (Fig. 1B).

**Figure 2:**
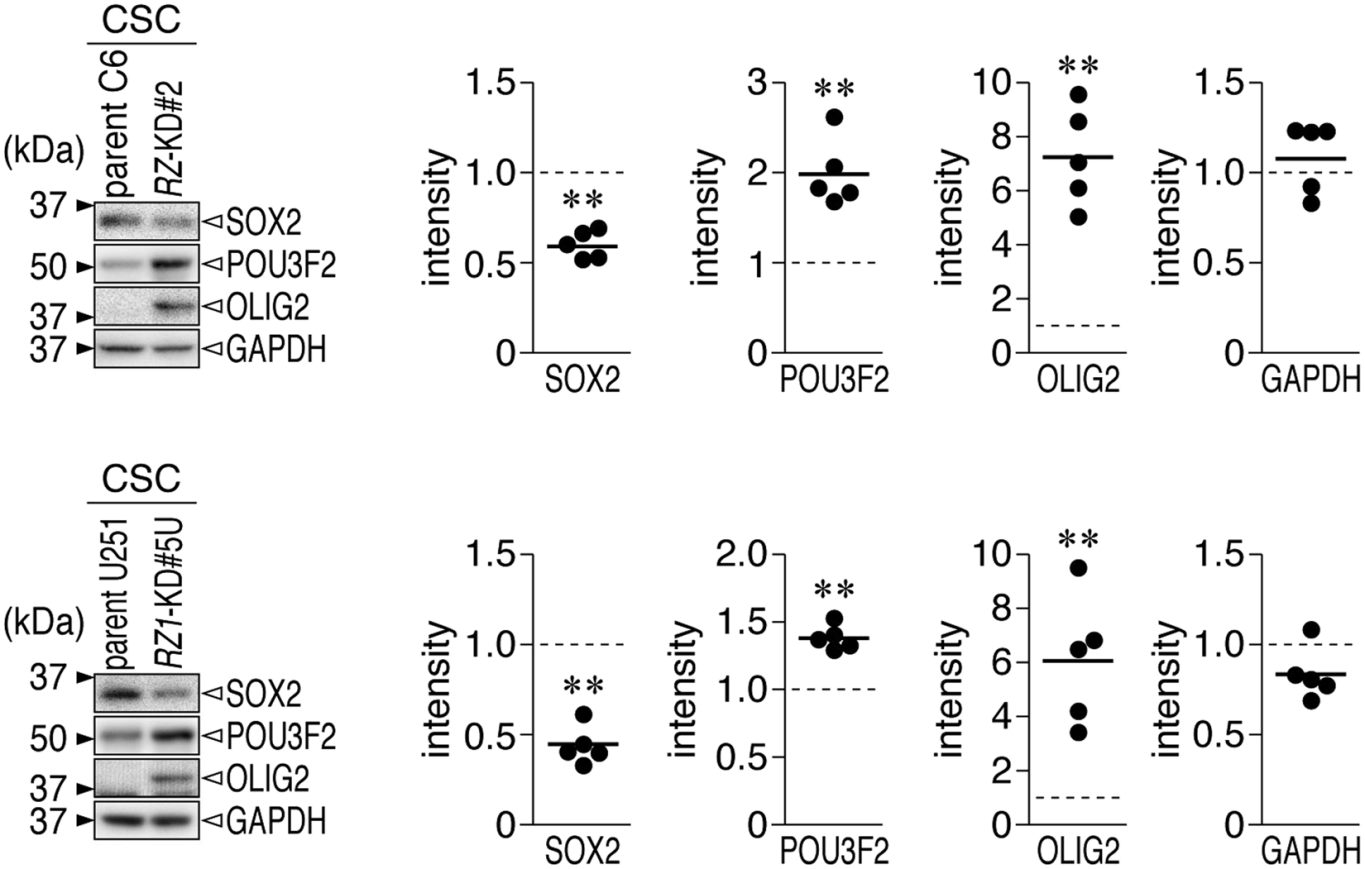
Altered expression of core transcription factors in *Ptprz*-knockdown glioblastoma cells. Western blots using antibodies against SOX2, OLIG2, POU3F2, and SALL2. Parental C6 and *RZ*-KD#2, and parental U251 and *RZ1*-KD#5U cells were cultured in CSC medium as in Fig. 1A. SALL2 proteins were not detected in C6 or U251 cells. Sample loading was verified by immunostaining with GAPDH. Images are representative of five independent cultures. The plots show the relative densitometric units of the staining intensity in the *PTPRZ*-knockdown cells and the staining intensity in the parental cells. *P < 0.05; **P < 0.01 (Student’s *t*-test). Full-length blots and gels are presented in Supplementary Fig. S6.

Next, we examined the effect of *PTPRZ* knockdown on the expression of the core transcription factors that are reportedly involved in sphere formation by glioblastoma cells and in reprogramming differentiated glioblastoma cells into stem-like states^25^. Under the sphere culture conditions, protein expression of SOX2 was decreased, whereas the expression of OLIG2 and POU3F2 was increased in *PTPRZ*-knockdown C6 and U251 cells compared with the corresponding parental cells (Fig. 2), suggesting a contribution by PTPRZ to stem cell signaling in glioblastoma cells. We found that these transcription factors were also expressed in C6 and U251 cells under normal culture conditions, and their expression was altered by the *PTPRZ* knockdown (Supplementary Fig. S1A).

**Figure 3:**
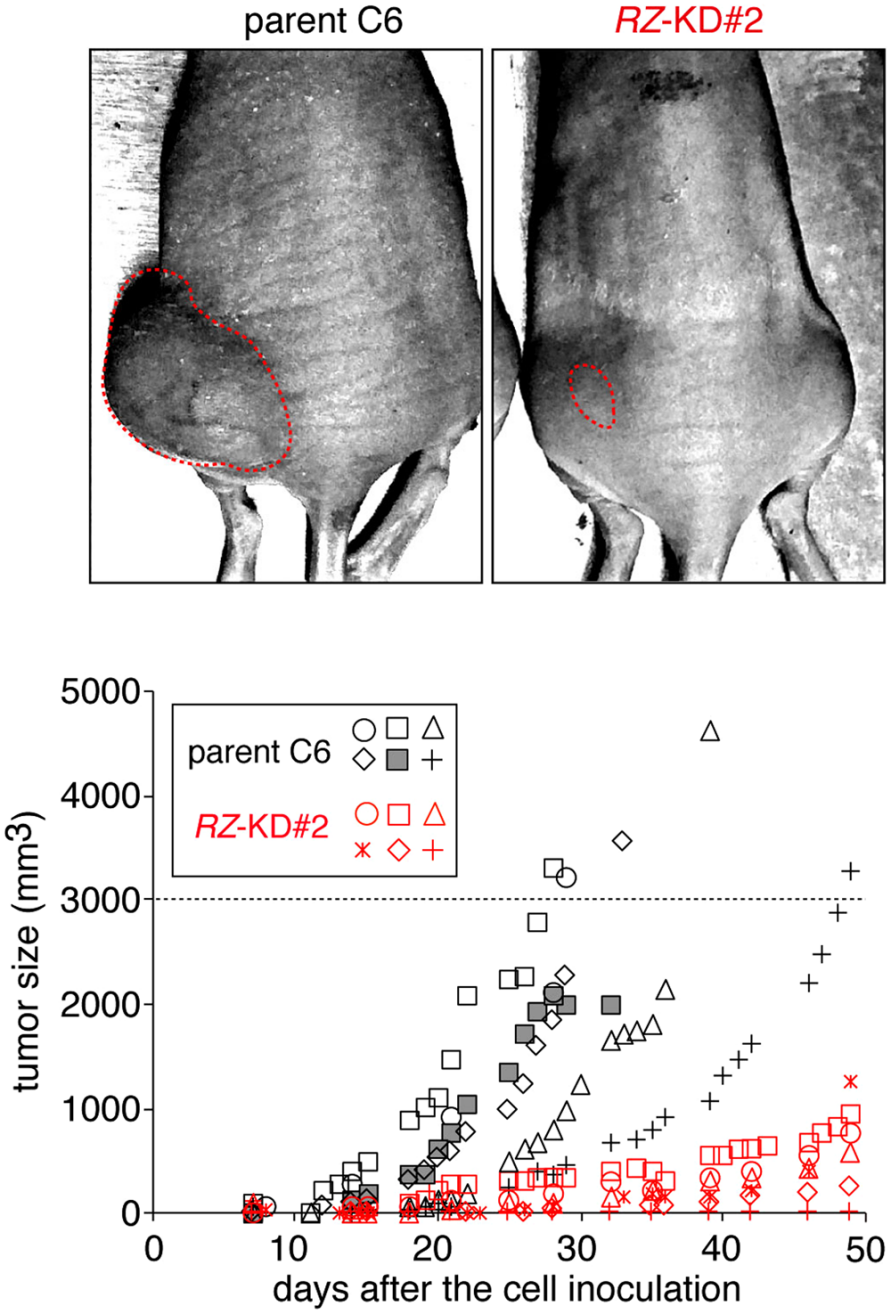
Decreased tumor growth in the *Ptprz*-knockdown glioblastoma cell xenografts. Subcutaneous xenograft tumor model. Nude mice were implanted subcutaneously with parental and *RZ*-KD#2 cells (5 × 10^6^ cells). The results were obtained from five independent cell preparations for the C6 group and two independent cell preparations for the *RZ*-KD#2 cell group. Images are representative of six animals per group 30 days after the cell injection, in which the tumor rims were surrounded by red dotted lines. Tumor size was measured until it reached the humane endpoint for sacrifice (>3,000 mm^3^ or 50 days after the cell injection): the C6 group reached the predetermined humane endpoint at 30 days after cell injections. The plot shows tumor growth for each animal. One mouse (a gray square symbol) was sacrificed because of tumor necrosis and bleeding at 34 days. The days required to reach a tumor volume of 3,000 mm^3^ were significantly different between the parental C6 and *RZ*-KD#2 groups (*P* < 0.01; Mann-Whitney *U*-test). The average tumor sizes at 30 days were significantly different between the two groups (*P* < 0.01, Student’s *t*-test).

We previously reported that the *RZ*-KD#2 clone produces intracranial tumors with a significantly slower growing rate than parental C6 cells 7 days after cell inoculation in syngeneic rats^6^. To evaluate the effects of *Ptprz* knockdown on tumorigenicity and stemness *in vivo*, we herein compared tumor development of the C6 and *RZ*-KD#2 cells via subcutaneous transplantation of the cells into nude mice during a period of over one month (Fig. 3). Parental C6 cells formed large tumors, whose volumes reached 3,000 mm^3^ by 50 days, which was a predetermined humane endpoint. In contrast, mice injected with *RZ*-KD#2 cells showed minimal tumor growth during the predetermined experimental period. Taken together, these results support the view that PTPRZ plays important roles in maintaining glioma stemness and tumorigenicity.

### Isolation of an allosteric inhibitor, NAZ2329, for R5 RPTPs

**Figure 4:**
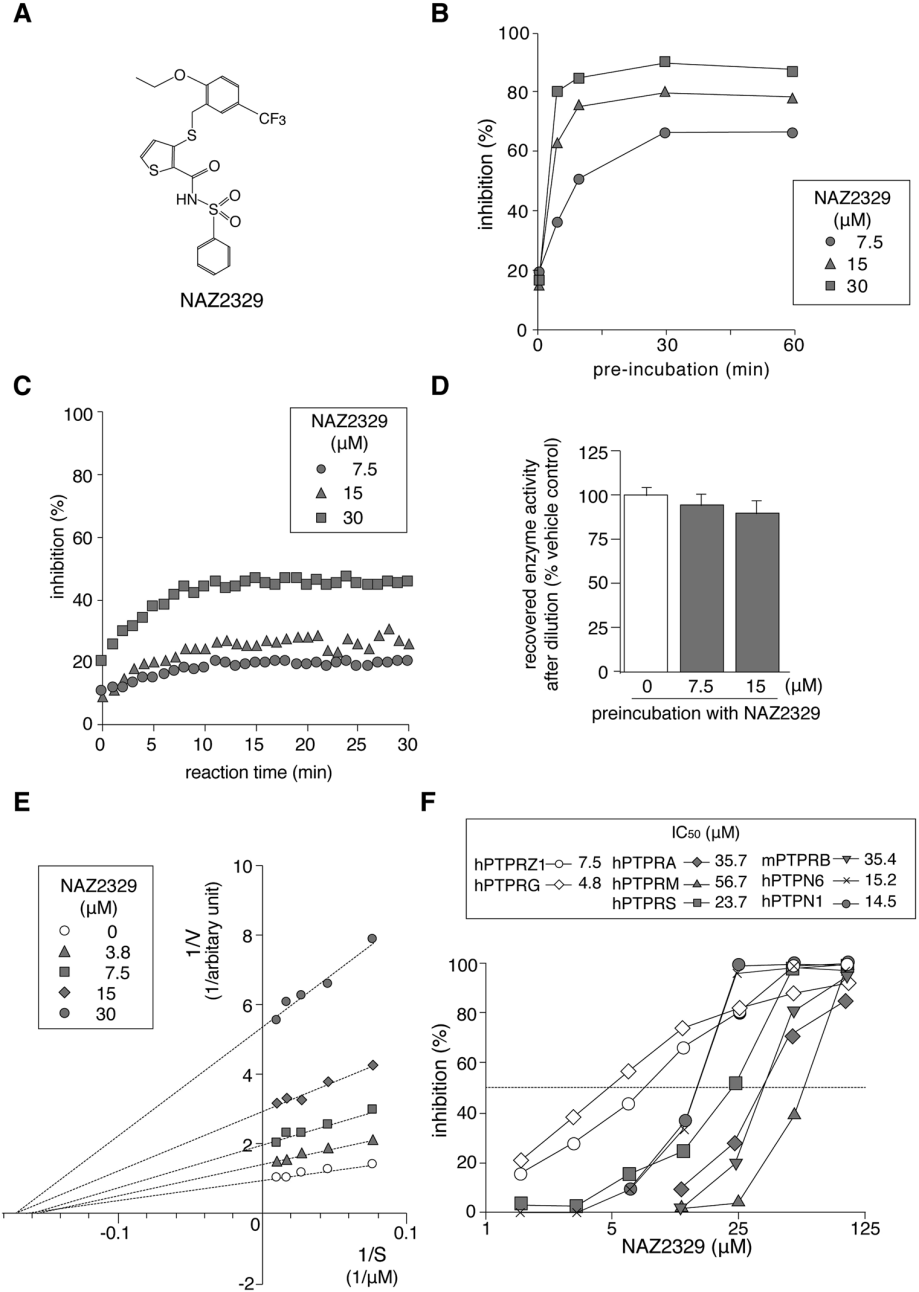
NAZ2329, an allosteric inhibitor of R5 RPTP subfamily members. (**A**) Structure of NAZ2329. (**B**) Effect of preincubation of NAZ2329 on its inhibitory activity against PTPRZ. Recombinant human PTPRZ1 enzyme (the whole intracellular region) was premixed with NAZ2329 for the indicated times and followed by addition of a fluorogenic DiFMUP (non-specific PTP substrate, 6,8-difluoro-4-methylumbiliferyl phosphate) to measure PTP inhibitory activity. (**C**) Time dependence. The PTPRZ1 enzyme was added to the inhibitor–substrate mixture, and the inhibitory activity was determined at each time point relative to the vehicle control. (**D**) Reversibility of inhibition. PTPRZ1 enzymes were preincubated with NAZ2329 at the indicated concentrations for 30 min. The mixture was then 20-fold diluted, and recovered PTP activity was expressed as the relative activity compared with the vehicle-treated enzyme. (**E**) Lineweaver-Burk plot analysis. PTPRZ1 enzymes were preincubated with NAZ2329 for 60 min, and the PTP activity was measured using DiFMUP. (**F**) Concentration-inhibition curves of NAZ2329 for representative PTP members, including human PTPRZ and PTPRG (another R5 RPTP subfamily member), human PTPRS (R2A subfamily), human PTPRM (R2B subfamily), mouse PTPRB (R3 subfamily), human PTPRA (R4 subfamily), human PTPN1 (non-transmembrane PTP, NT1 subfamily), and human PTPN6 (NT2 subfamily), were generated. Assays were performed using DiFMUP. IC_50_ values obtained are shown in the inset. Data represent the averages of two or three separate experiments.

We previously reported SCB4380 as the first small molecule inhibitor to be identified for PTPRZ, using a high-throughput screen^6^. However, this compound was cell impermeable^6^. We have newly identified NAZ2329, 3-{[2-ethoxy-5-(trifluoromethyl)benzyl]thio-*N*-(phenylsulfonyl)thiophene-2-carboxamide (Fig. 4A), as a cell-permeable inhibitor from other hits found in our previous screening^6^. NAZ2329 has a molecular weight (MW) of 501.6 with the *n*-octanol/water partition coefficient (logP) = 5.15, total polar surface area (TPSA) = 72.47 Å^2^, number of H-bond donors (HBD) = 1, number of H-bond acceptors (HBA) = 5, and rotatable bonds = 9; these values were obtained using an online calculator (molinspiration.com). This compound partially fulfills Lipinski’s rule (MW ≤ 500, LogP ≤ 5, HBD ≤ 5, HBA ≤ 10) (ref. 30) but suitably fulfills Veber’s rule (rotatable bonds ≤ 10, TPSA < 140 Å^2^, (or HBA + HBD ≤ 12) (ref. 31) for the prediction of bioavailability.

We found that preincubation of the enzyme proteins with NAZ2329 resulted in a time-dependent increase in the inhibitory effect of NAZ2329 on the catalytic activity of PTPRZ1, which increased until 30 min and remained constant thereafter (Fig. 4B). The inhibitory activity gradually increased during 30 min in the case without preincubation (Fig. 4C). However, it did not reach the maximal level observed with the preincubation (compare with Fig. 4B). After a 20-fold dilution of the enzyme-compound mixture with the reaction buffer, the enzymatic activity was fully recovered to the control level, indicating a reversible inhibition property of NAZ2329 (Fig. 4D). These results suggest that pre-binding to apo-PTPRZ1 is necessary for its maximum inhibition. Therefore, we conducted enzyme assays by employing a 60-min preincubation period. NAZ2329 showed a noncompetitive-type inhibition (Fig. 4E). Concentration-dependent inhibition curves revealed that NAZ2329 preferentially inhibited PTPRZ1 and PTPRG over the other PTPs tested in the 3~10 µM concentration range (Fig. 4F).

**Figure 5:**
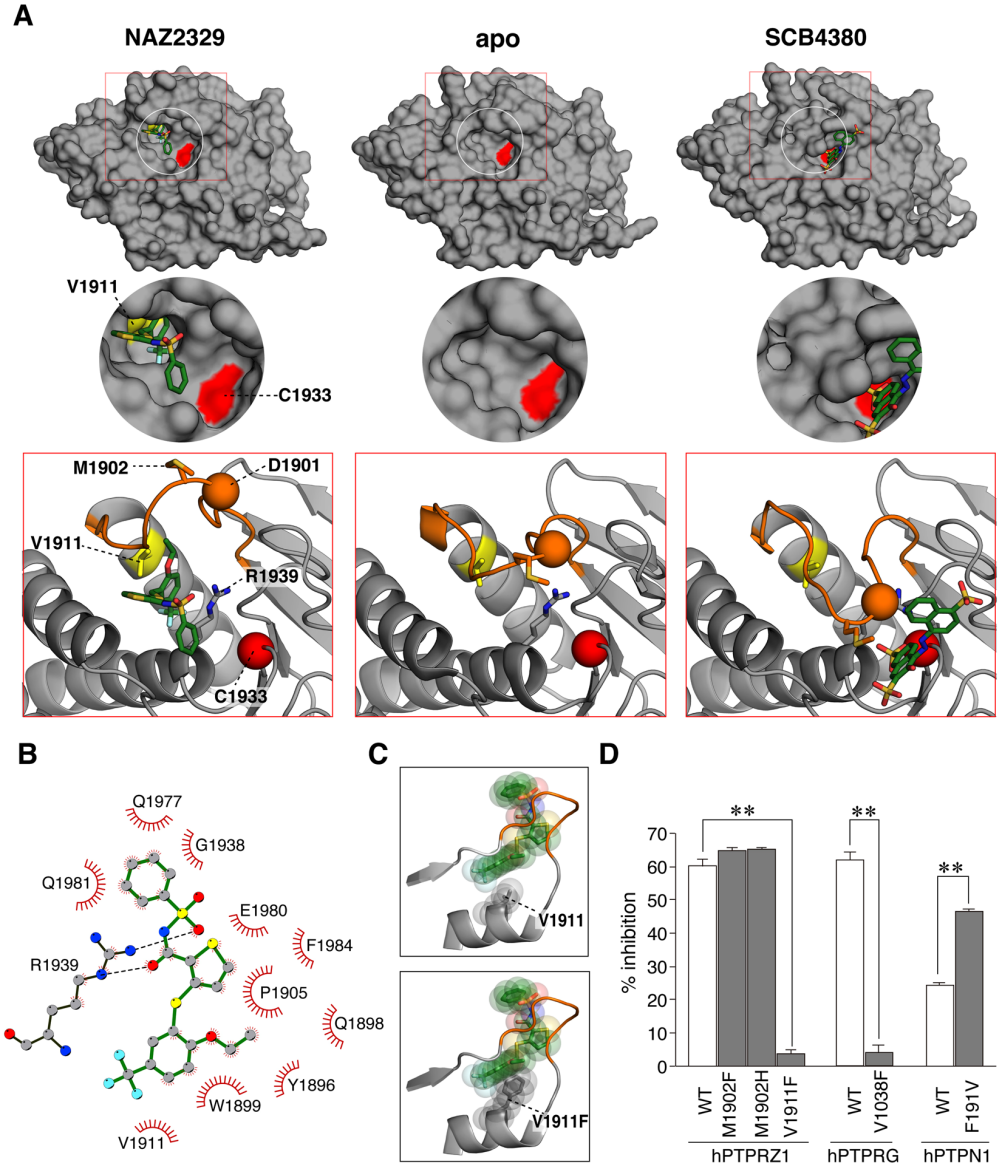
Structural basis for PTPRZ inhibition of NAZ2329. (**A**) An enlarged view of the X-ray structure of human PTPRZ1-D1 complexed with NAZ2329 (PDB ID: 5H08) (left), X-ray structure of the apo (open) form of PTPRZ1-D1 (PDB ID: 5AWX, ref. 6, middle) and a computer-modeled structure of the closed form of PTPRZ1-D1 complexed with a competitive inhibitor, SCB4380 (ref. 6, right), are shown. PTPRZ1-D1 is shown as a gray surface representation, in which Cys1933 at the active site and Val-1911 at the bottom of the allosteric pocket are indicated in red and yellow, respectively. Inhibitors are colored according to the atomic species in a stick figure representation as follows: oxygen (red), nitrogen (blue), sulfur (yellow), and carbon (green). The bottom figures show the conformation of the WPD loop (orange) as cartoon representations, in which Asp1901 and Cys1933 are indicated by orange and red spheres, respectively. Side chains of Arg1939 at the active site, Met1902 in the WPD loop, and Val-1911 at the bottom of the allosteric binding area are also shown in the stick representation. (**B**) LigPlot representation of the NAZ2329 interaction with PTPRZ1-D1. The predicted hydrogen bonds (broken black lines) and residues (red spoked arcs) involved in the hydrophobic interaction are shown. (**C**) Predicted steric hindrance toward NAZ2329 binding after substitution of Val-1911 with a bulky Phe residue. The wild-type (upper) and replaced (lower) structures of PTPRZ1-D1 are shown in stick and transparent-sphere models. The structure was built by direct replacement of Val-1911 by Phe using the mutagenesis tool in the PyMOL software. (**D**) Inhibitor sensitivity to point substitution mutants of PTPs. Inhibition by 10 µM NAZ2329 is presented as a percentage of the DMSO control for each enzyme (mean ± S.E. of three separate experiments). **P < 0.01, significantly different from the wild-type enzyme (Student’s *t*-test).

NAZ2329 showed a more potent inhibition of the first PTP domain (PTPRZ1-D1 fragment) (IC_50_ of 1.1 µM) than the whole intracellular (D1 + D2) fragment (IC_50_ of 7.5 µM), indicating that the inhibitory effect of this compound is caused by its binding to the active D1 domain (Supplementary Fig. S2). To further clarify the inhibitory mechanism of NAZ2329, we obtained complex crystals containing PTPRZ1-D1 and NAZ2329 by soaking them with PTPRZ1-D1 crystals^6^ and elucidated their structures at a resolution of 2.53 Å (Fig. 5A, left and Supplementary Table. S1). NAZ2329 was trapped by a newly identified cleft just under the catalytic WPD loop. The catalytic WPD loop with NAZ2329 (Fig. 5A, left) showed an extraordinarily open conformation compared with that of the apo structure (Fig. 5A, middle). In contrast, the docking structure with SCB4380, a competitive inhibitor for PTPRZ (ref. 6), showed a closed conformation for the WPD loop (Fig. 5A, right). Stabilization of the aberrantly opened conformation of the WPD loop by NAZ2329 suggests that NAZ2329 interrupts the transition from the open WPD conformation to the closed WPD conformation that is essential for the catalysis to occur^32^.

NAZ2329 formed two hydrogen bonds with the catalytically essential Arg-1939 in the conserved PTP loop^10^ (Fig. 5B), which may prevent the bidentate interaction between the arginine side chain and phosphate group of the substrate, thereby inhibiting enzymatic hydrolysis^10^. Met-1902, at the hinge of the WPD loop, has been identified as a unique residue in the R5 RPTP members^10^. We first speculated that Met-1902 may contribute to the more open WPD conformation. However, the substitution of Met-1902 with Phe (in PTPN1, PTPN2, PTPRA, and PTPRE) or His (in many other PTPs) (ref. 10) did not affect the sensitivity to the inhibitor (Fig. 5D, M1902F and M1902H).

We then focused our attention on Val-1911, which lies at the bottom of the newly identified cleft (Fig. 5C). It was conserved in PTPRG, but considerable diversity was found in the other PTPs (see Supplementary Fig. S3A). It was presumed that the substitution of Val-1911 in PTPRZ1 with the bulky Phe, as in PTPN1, may cause steric hindrance in the binding of NAZ2329 (Fig. 5C). Indeed, the V1911F substitution markedly reduced the sensitivity to NAZ2329 (Fig. 5D). Like PTPRZ1, the V1038F mutation in PTPRG resulted in resistance to NAZ2329, but, inversely, the F191V mutant of PTPN1 became more sensitive than the wild-type PTPN1 (Fig. 5D, ~2-fold change at 10 µM). Importantly, the V-to-F substitution in PTPRZ and PTPRG hardly affected their sensitivities to competitive inhibitors, such as SCB4380 and vanadate (Supplementary Fig. S3B, only ~5% change on average). All these results support an allosteric inhibition mechanism by NAZ2329.

### Cellular effects of NAZ2329

**Figure 6:**
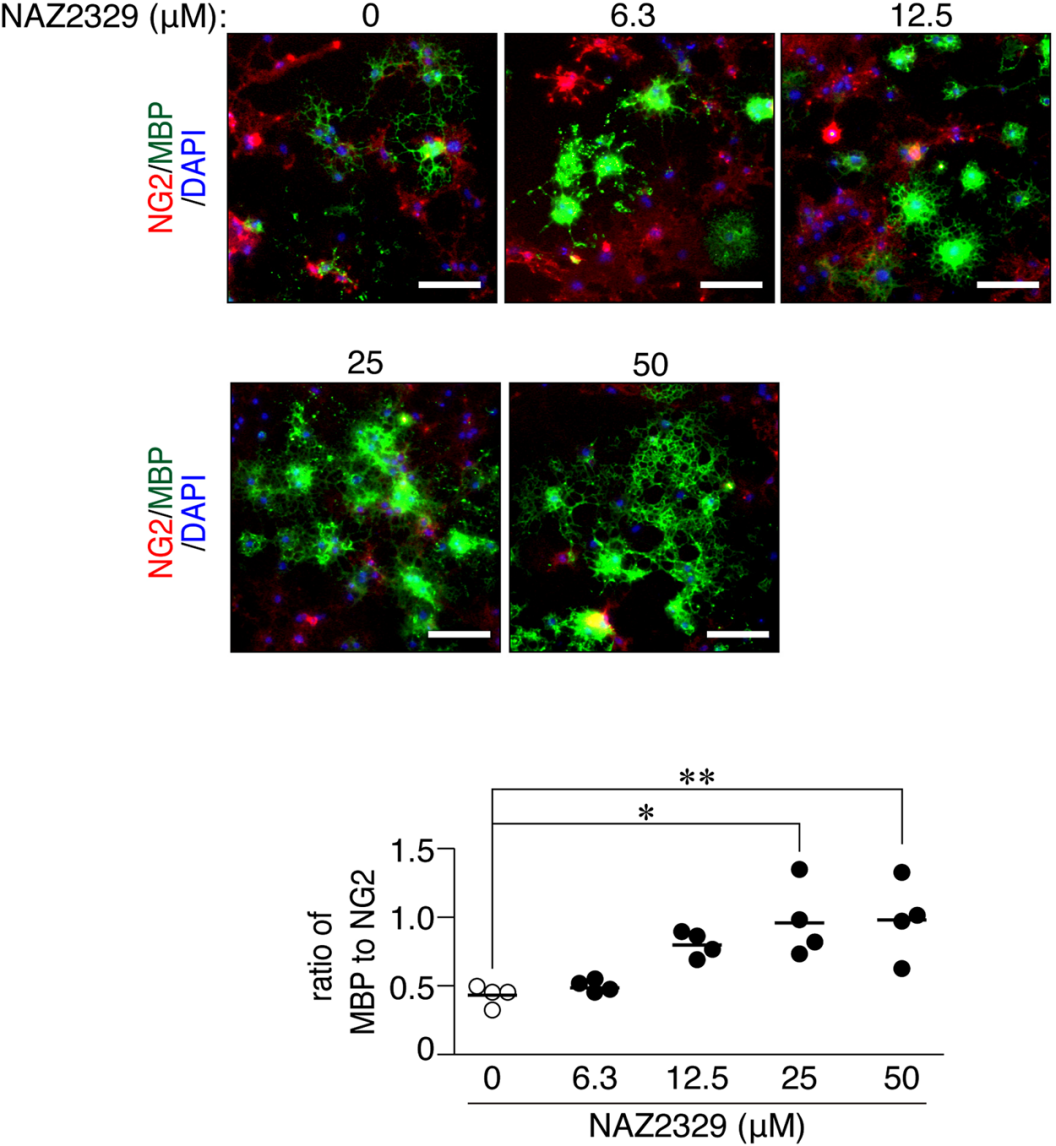
Effects of NAZ2329 treatment on oligodendrocyte differentiation. Mouse oligodendrocyte-lineage OL1 cells were cultured in differentiation media containing the indicated concentrations of NAZ2329. After 10 days, cells were fixed with formalin and stained with anti-NG2 proteoglycan (oligodendrocyte precursor cells, red) and anti-MBP (oligodendrocyte, green) antibodies, in conjunction with the DAPI-labeling of nuclei (blue). Scale bars, 100 µm. The plot shows the ratio of MBP-positive cells to NG2-positive cells, in which each dot corresponds to an independent cell culture (*n* = 4 each). *P < 0.05; **P < 0.01, significantly different from vehicle-treated cells (one-way ANOVA with Bonferroni *post hoc* tests).

The catalytic activities of PTPRZ receptor isoforms play important roles in maintaining oligodendrocyte precursor cells in an undifferentiated state^14, 15^. The endogenous inhibitory ligand of PTPRZ, pleiotrophin, induces oligodendrocyte differentiation by binding to the extracellular region of PTPRZ and inducing dimer or oligomer formation by this receptor^14–16^. Like pleiotrophin, NAZ2329 dose-dependently induced the differentiation of oligodendrocyte precursor OL1 cells to mature oligodendrocytes (Fig. 6), indicating the cell permeable property and non-toxic nature of this compound.

**Figure 7:**
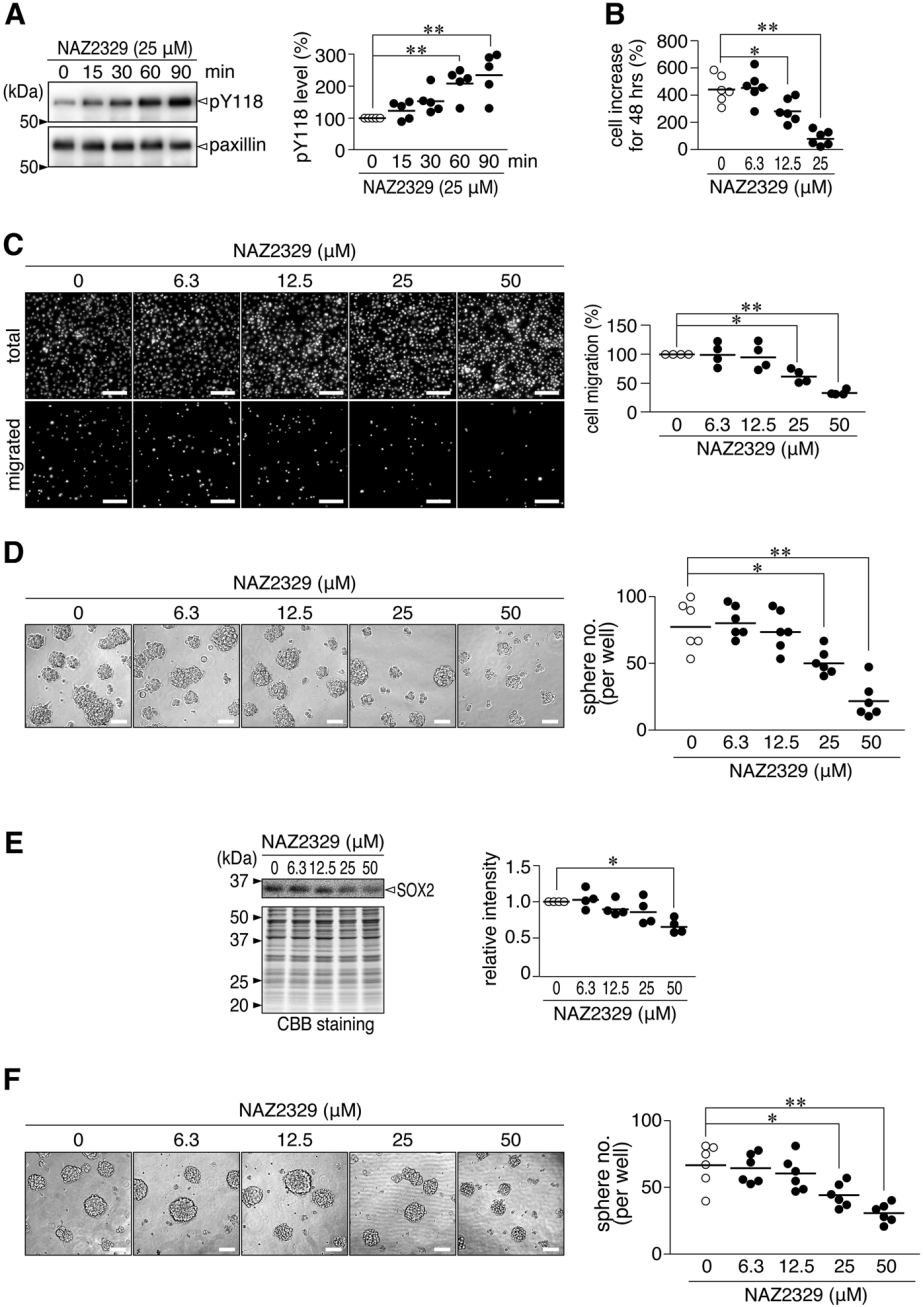
Cellular effects of NAZ2329 on the malignant phenotypes of C6 cells. (**A**) Phosphorylation of paxillin at Tyr-118. C6 cells were incubated with NAZ2329 for the indicated periods. Immunoprecipitated paxillin was analyzed by Western blotting using anti-pY118-paxillin and anti-paxillin. Blots are representative of five independent cultures. The plot shows the intensity of pY118 staining relative to the paxillin level, normalized to the vehicle control in each experiment. **P < 0.01, significantly different from the vehicle by one-way ANOVA with Bonferroni *post hoc* tests). (**B**) Cell proliferation assay. C6 cells were incubated for 48 h with NAZ2329 in normal medium containing 2% FBS. The plot shows the percentage increase in the cell number. *P < 0.05, **P < 0.01 (one-way ANOVA with Bonferroni *post hoc* tests). (**C**) Boyden chamber assay. Cells were allowed to migrate for 3 h. DAPI-stained nuclei are shown before (total) and after (migrated) the removal of cells remaining in the top chamber. Scale bars, 100 µm. The plot shows the migrated cell number normalized to the vehicle. *P < 0.05, **P < 0.01 significantly different from the vehicle (one-way ANOVA with Bonferroni *post hoc* tests). (**D**,**E**) Sphere formation (**D**) and SOX2 expression (**E**). C6 cells were cultured in CSC medium for 7 days with indicated concentrations of NAZ2329. Images are representative of five independent cultures. Scale bars, 100 µm. The plots show the sphere number per well (D) and staining intensity of SOX2, normalized to the vehicle (**E**). *P < 0.05, **P < 0.01 significantly different from the vehicle (one-way ANOVA with Bonferroni *post hoc* tests). (**F**) Self-renewal of C6 spheres. C6 spheres were initially developed in CSC medium for 7 days, followed by an incubation with NAZ2329 in CSC medium for 5 days. Images are representative of five independent cultures. Scale bars, 100 µm. The plot shows the sphere number per well. *P < 0.05, **P < 0.01 (one-way ANOVA with Bonferroni *post hoc* tests). Full-length blots and gels are presented in Supplementary Fig. S7.

The treatments of the C6 cells with NAZ2329 significantly enhanced the phosphorylation level of paxillin at Tyr-118, a PTPRZ substrate site^33^ (Fig. 7A). In addition, NAZ2329 inhibited cell proliferation (Fig. 7B) and migration (Fig. 7C) in C6 cells, similar to the knockdown of *Ptprz* (ref. 6). Notably, NAZ2329 dose-dependently inhibited sphere formation by C6 cells (Fig. 7D), which was accompanied by a decrease in SOX2 expression (Fig. 7E). Moreover, NAZ2329 suppressed self-renewal of sphere-forming C6 cells (Fig. 7F). NAZ2329 exerted similar effects in human U251 cells (Supplementary Fig. S4).

**Figure 8:**
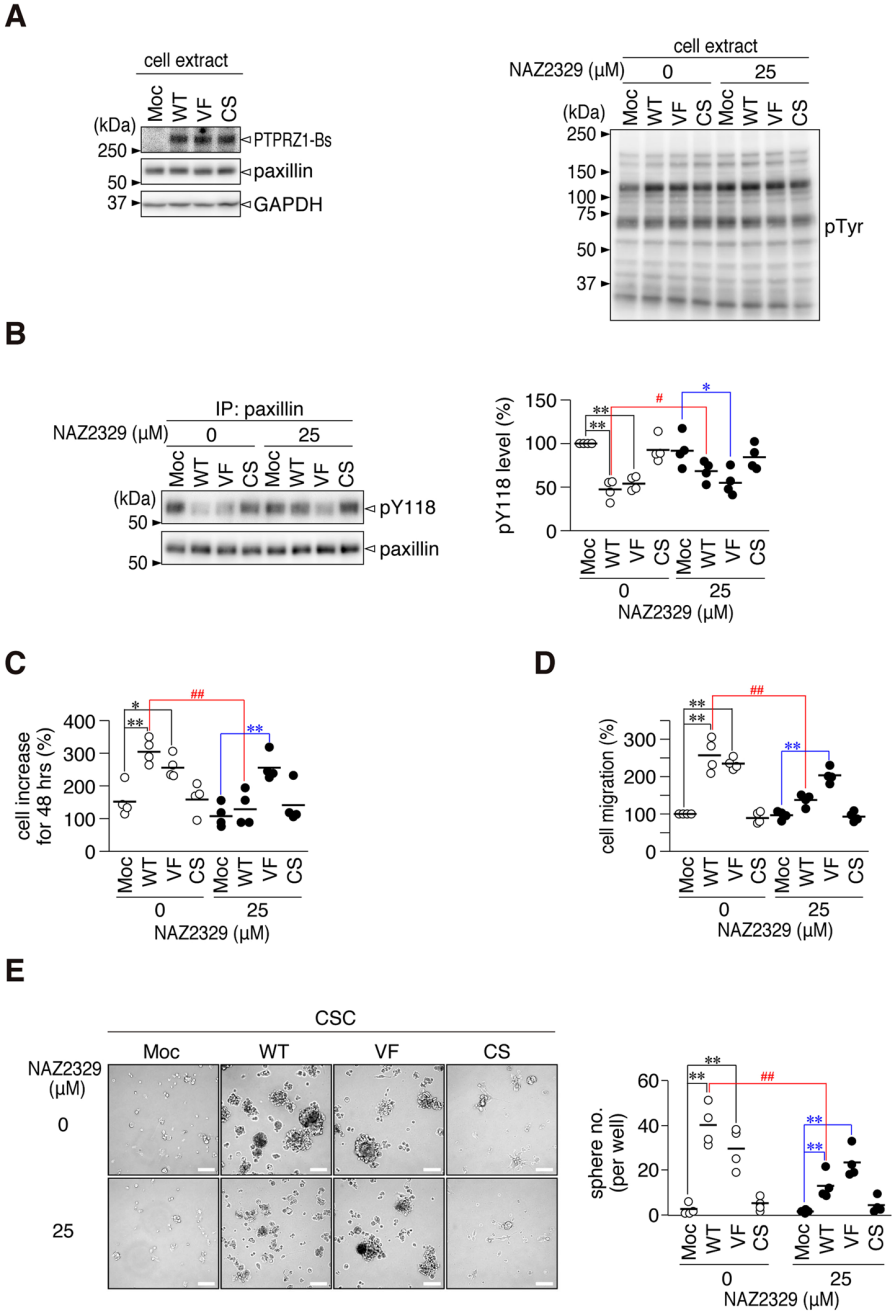
Inhibitory effects of NAZ2329 on PTPRZ activity in C6 cells. (**A**,**B**) Protein expression of PTPRZ-1B and paxillin (A, left), the overall tyrosine (Tyr)-phosphorylation pattern of cellular proteins (A, right), and the Tyr-phosphorylation levels of paxillin at Tyr-118 (**B**). Sample loading was verified by immunostaining with GAPDH. *RZ*-KD#2 cells transfected with an expression plasmid for wild-type (WT) PTPRZ1-B, the V1911F (VF) or C1933S (CS) mutants of human PTPRZ1-B, or empty plasmid (Moc) were treated with 25 µM NAZ2329 or vehicle for 60 min, and the cell extracts were subjected to analyses by Western blotting. Blots are representatives of four independent experiments. Paxillin proteins were immunoprecipitated, and their Tyr-phosphorylation levels at Tyr-118 were analyzed as in Fig. 7A: pY118 levels were normalized to the vehicle-treated mock cells in each experiment. (**C** to **E**) Cell proliferation (**C**, 48 h), cell migration (**D**, 3 h), and sphere formation (**E**) in *RZ*-KD#2 cells transfected with the indicated construct were analyzed as in Fig. 7B,C and D, respectively. Scale bars, 100 µm. Statistical analyses of the data were performed as follows: *P < 0.05; **P < 0.01, significantly different from the mock-transfected cells in each treatment group (vehicle or NAZ2329) (one-way ANOVA with Bonferroni *post hoc* tests). ^#^ *P* < 0.05; ^##^ *P* < 0.01, significantly different between the vehicle and NAZ2329 treatment (Student’s *t*-test in each transfectant). The blots and images are representative of four independent experiments, and their full-length blots (**A** and **B**) and representative microscopic images (**D**) are presented in Supplementary Fig. S8.

**Figure 9:**
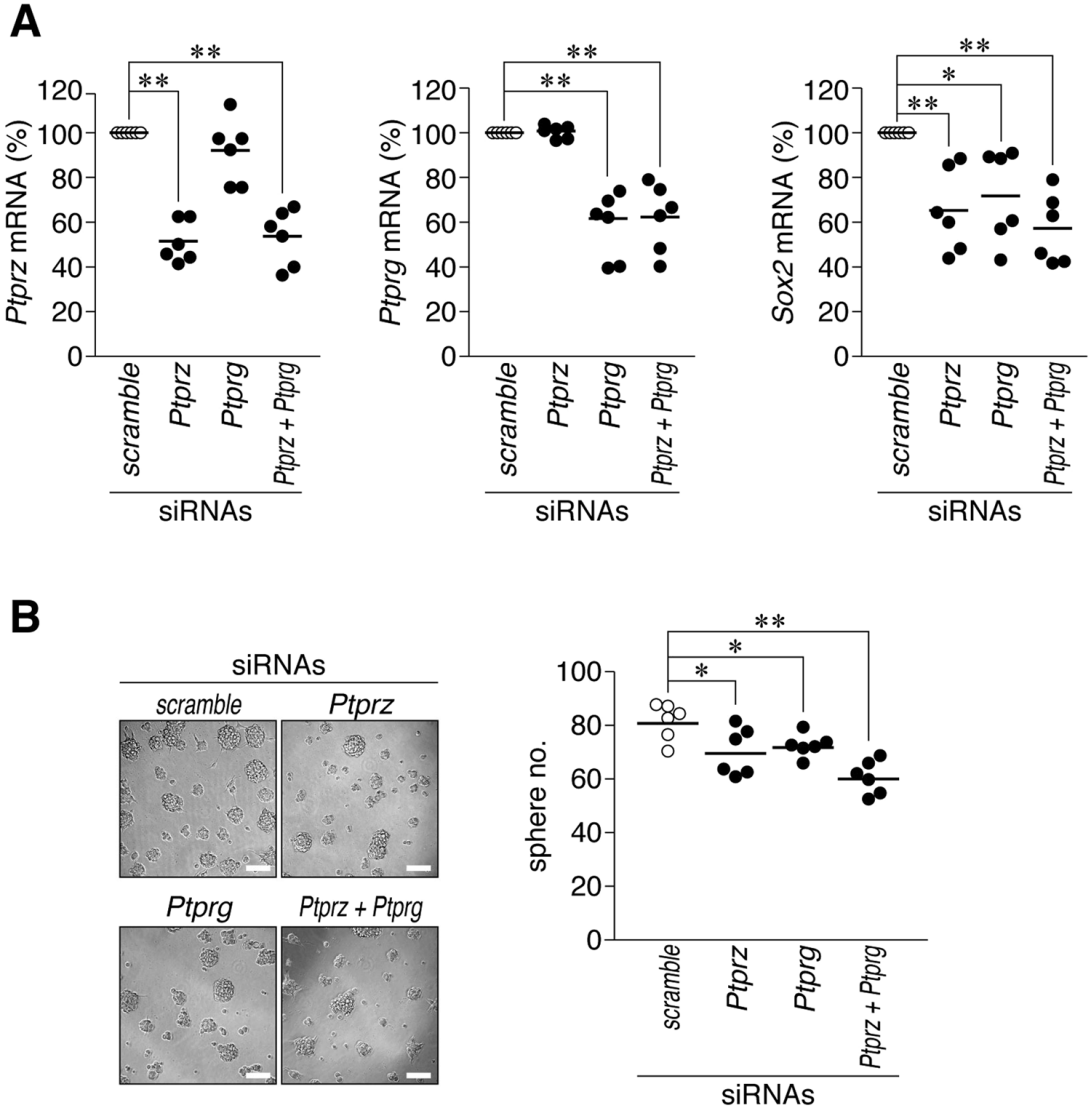
Effects of *Ptprg* knockdown on C6 stem cell-like properties. (**A**) Quantitative RT-PCR analyses. C6 cells were electroporated with short interfering RNA (siRNA) for rat *Ptprz*, *Ptprg*, or *Ptprz* plus *Ptprg*. After a 48-h culture, RNA was extracted from cells and subjected to quantitative RT-PCR. The plots show the mRNA expression of *Ptprz*, *Ptprg*, and *Sox2* normalized to *Gapdh* expression. (**B**) Sphere formation assay. Cells transfected with the indicated siRNAs were cultured for 7 days in CSC medium. Scale bars, 100 µm. Images are representative of six independent cultures. The plot shows sphere number per well. *P < 0.05, **P < 0.01, significantly different from the scramble control (Student’s *t*-test).

We then performed phenotypic rescue experiments in *Ptprz*-knockdown C6 cells. To avoid targeting by the shRNA that was directed against the coding region of the rat *Ptprz* (ref. 6), we prepared expression constructs using human *PTPRZ1* for these experiments. Expression of wild-type PTPRZ1-B or the V1911F mutant of PTPRZ1-B (NAZ2329-resistant and phosphatase active mutant) in *RZ*-KD#2 cells rescued the effects of the PTPRZ knockdown on paxillin phosphorylation (Fig. 8A and B), cell proliferation (Fig. 8C), cell migration (Fig. 8D), and CSC formation (Fig. 8E). In contrast, no effects were observed with the forced expression of the PTPase-inactive C1933S mutant. NAZ2329 interfered with the rescue effect by the wild-type, but not by the V1911F mutant (Fig. 8A to E); this was consistent with the finding that NAZ2329 showed a lower inhibitory effect on the catalytic activity of PTPRZ1-V1911F *in vitro* (see Fig. 5D). We also examined the effect of *PTPRG* knockdown by siRNA. *Sox2* expression and sphere formation were concomitantly decreased in C6 cells by the knockdown of *Ptprg* and *Ptprz* (Fig. 9). As NAZ2329 preferentially inhibits both PTPRG and PTPRZ1 (see Fig. 4F), NAZ2329 is expected to be superior to sole inhibitors of PTPRZ or PTPRG for inhibiting the malignant properties of glioblastoma cells.

### Antitumor effects of NAZ2329

**Figure 10:**
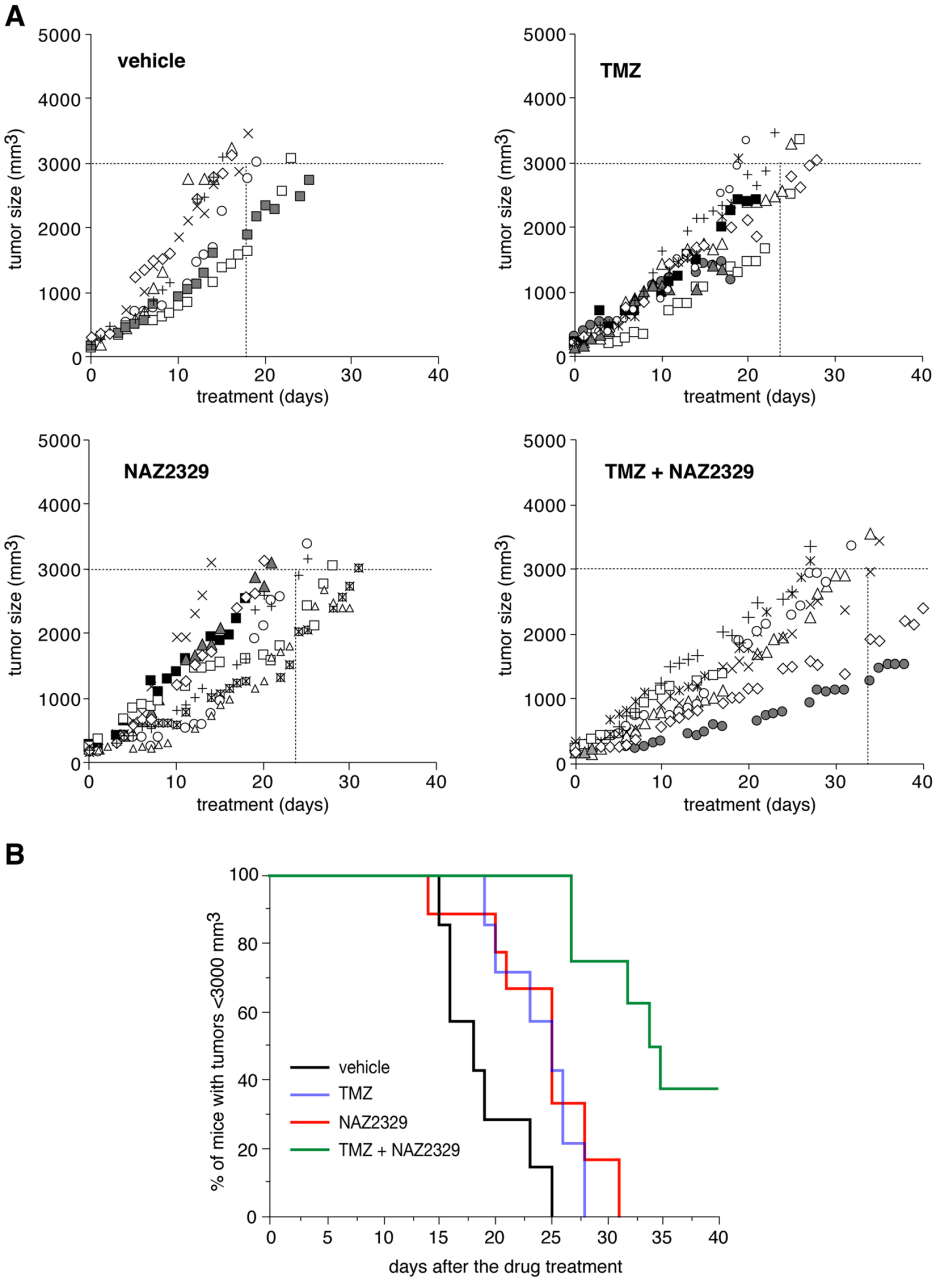
Antitumor effects of NAZ2329 on the C6 mouse xenograft model. (**A**) Nude mice were subcutaneously implanted with C6 cells (5 × 10^6^ cells), and tumor size was monitored until the criteria were met (>150 mm^3^). At this time, mice were randomly divided into 4 treatment groups. DMSO as the vehicle control (*n* = 7), NAZ2329 (45 µmol (22.5 mg)/kg body weight, *n* = 9), temozolomide (TMZ, 50 mg/kg, *n* = 9), and the combination of NAZ2329 (45 µmol/kg) with TMZ (50 mg/kg) (*n* = 9) were administered intraperitoneally twice per week until the humane endpoint (>3,000 mm^3^ tumor size or 40 days after the treatment). Tumor growth in each animal and the number of days required to reach a tumor volume of 3,000 mm^3^ are shown in the graph. Five mice were sacrificed due to tumor necrosis (shown by gray symbols in the graph), and two mice unexpectedly died suddenly during the observation period (black symbols in the graph). (**B**) A Kaplan-Meier analysis of the four treatment groups shown in *A*. Significant differences were observed in the days until the tumor volume reached 3,000 mm^3^ between the vehicle vs NAZ2329/TMZ (*P* < 0.05), NAZ2329 vs NAZ2329/TMZ (*P* < 0.05) and TMZ vs NAZ2329/TMZ (*P* < 0.05) (Kruskal-Wallis test followed by Steel-Dwass test).

We evaluated the antitumor effects of NAZ2329 in the C6-xenograft nude mouse model. The cytotoxic anticancer alkylating agent, temozolomide (TMZ), is the most widely used drug for the treatment of brain tumors, including malignant gliomas^24^. We verified that TMZ showed no significant inhibitory effects on PTPRZ activity (Supplementary Fig. S2) and then assessed the combined effects of TMZ and NAZ2329. Each mouse was randomly placed into one of four groups, and the treatment started when tumor sizes reached 150 mm^3^. Comparisons of the tumor growth curves indicated that the combination of NAZ2329 with TMZ had the apparent effect of a stronger delay in tumor growth than for NAZ2329 or TMZ alone (Fig. 10), where the median day for the tumor size to reach 3,000 mm^3^ was defined as the predetermined endpoint. The data obtained were 34 days for the combination of NAZ2329 with TMZ, 25 days for NAZ2329 alone, 25 days for TMZ alone, and 17 days for the DMSO vehicle. Considering the IC_50_ values in Fig. 4F, we cannot exclude contributions by the inhibitory effects of NAZ2329 on other PTPs such as PTPN1 in this experiment. Taken all together; however, it is possible to postulate that selective inhibitors of the R5 RPTP subfamily members, PTPRZ and PTPRG, may be applicable to a differentiation-inducing therapy for malignant gliomas.

## Discussion

Several groups including ours have already shown that knockdown^6, 22^, antibody-mediated blockade^23^, or pharmacological inhibition^6^ of PTPRZ reduces the aggressive proliferation and migration properties of glioblastoma cells. In the present study, we demonstrated that PTPRZ and its family member PTPRG play critical roles in maintaining the stem cell-like properties of glioblastoma cell lines. NAZ2329, the first cell-permeable inhibitor of the R5 RPTPs, suppressed the stem cell-like properties and the strong proliferation and migration abilities of the glioblastoma cells. We employed a subcutaneous xenograft model to evaluate the effects of the NAZ2329 treatment on tumor growth using C6 glioblastoma cells, which have been reported to be highly resistant to temozolomide (TMZ) (ref. 34). The synergistic antitumor effects of NAZ2329 and TMZ in the C6 glioblastoma xenograft model suggest that the combined treatment of TMZ with the R5 RPTP inhibitors is promising as a treatment for malignant gliomas.

The NAZ2329 structure slightly resembles those of the PTPRG inhibitors, 3-(3,4-dichlorobenzylthio) thiophene-2-carboxylic acid (compound 1) and its derivatives (ref. 35). However, the inhibition by compound 1 is reportedly a competitive-type inhibition (ref. 35). Moreover, these compounds failed to show inhibitory effects in the cellular context, probably due to the poor membrane permeability^35^. On the other hand, NAZ2329 showed a non-competitive-type inhibition (Fig. 4E) that was effective in cultured cells (Figs 6 to 9) and xenograft tumors (Fig. 10). NAZ2329 was trapped in a newly identified cleft just below the catalytic WPD loop. Thus, an inactive open conformation was considered to be fixed (Fig. 5A. left). Val-1911, which lies at the bottom of the pocket, is a non-conserved residue in the PTP family (Supplementary Fig. S3A). Substitution of Val-1911 in PTPRZ1 or Val-1038 in PTPRG to a bulky Phe reduces sensitivity to NAZ2329, probably through steric hindrance that affects the compound’s ability to enter the pocket (Fig. 5C and D).

NAZ2329 successfully suppresses the cell proliferation and cell migration of the differentiated glioblastoma cells, as well as cell sphere formation (Figs 7 to 8). NAZ2329 is the first cell-permeable R5 RPTP inhibitor. However, the average NAZ2329 levels in the plasma and brains of the C57BL6 mice 1 h after intraperitoneal injections of 30 mg/kg were 22 µg/ml and 0.3 µg/g (average values of two female mice), respectively, suggesting the poor BBB permeability of NAZ2329. Nevertheless, NAZ2329 is a potentially valuable lead therapeutic for the development of drugs that function through allosteric inhibitory mechanisms against PTPs, such as PTPRZ and PTPRG.

PTPs have long been recognized as “undruggable” targets because the phosphotyrosine mimetic properties of PTP inhibitors show poor cell permeability^5^. However, the recent discovery of allosteric inhibitors of PTP1B and SHP2 indicates that PTP-targeted drugs are promising candidates for anticancer therapy^4^. Trodusquemine (MSI-1436), an allosteric inhibitor of PTP1B, has been shown to suppress tumorigenesis in xenografts and abrogate metastasis in the NDL2 breast cancer mouse model^8^. This compound is currently under clinical development as a therapeutic candidate for HER2-positive breast cancer (Clinical trial registry no; NCT02524951). The orally bioavailable SHP2 inhibitor, SHP099, has more recently been developed^9^. SHP099 stabilizes SHP2 in an autoinhibited conformation and blocks oncogenic activation of SHP2-RAS–ERK signaling, thereby inhibiting cancer cell proliferation and tumor propagation in xenograft models^9^.

PTPRZ is strongly expressed in malignant gliomas^20, 21^, and the expression levels of *PTPRZ1* transcripts are reportedly associated with cancer stemness in primary human glioblastomas^26^. In glioblastoma cells, PTPRZ-B isoforms are predominant, and their expression levels show an increase in sphere-forming cells (Fig. 1B). The results of the present study revealed that the knockdown or inhibition of PTPRZ suppresses the stem-like cell properties of C6 and U251 glioblastoma cells (Figs 1, 2, 7 and 8), and therefore may induce their differentiation. Suvà, ML *et al*. reported that four core transcription factors (SOX2, OLIG2, POU3F2, and SALL2) reprogram differentiated glioblastoma cells into tumor-propagating stem-like cells^25^. All four factors co-bind large numbers of distal regulatory elements in a subset of stem-like tumor-propagating cells, though SOX2 and POU3F2 can each partially reprogram to induce spherogenic growth^25^. Notably, PTPRZ1 ranks second among 325 putative direct targets of these core transcription factors and is inferred to be targeted by SOX2, OLIG2, and POU3F2 (see Supplementary Table S3 in ref. 25). We herein found that expression of SOX2 was decreased, whereas expression of OLIG2 and POU3F2 were increased in both rat and human glioblastoma cell lines under sphere culture conditions (Fig. 2). PTPRZ is considered to be one of the direct downstream effectors of these core transcription factors, and their expression levels may be affected by PTPRZ activity.

It will be important to identify the signals that are downstream of PTPRZ and PTPRG that are involved in the regulation of stemness in CSCs. We have already identified and reported several PTPRZ substrates, including paxillin (refs 6, 33 and 36), G protein-coupled receptor kinase-interactor 1 (GIT1) (refs 33, 37 and 38), GTPase-activating protein for Rho GTPase (p190RhoGAP) (refs 14–16 and 39), and membrane-associated guanylate kinase, WW and PDZ domain-containing 1 (MAGI1) (refs 33 and 38), which are related to the migration/invasion and proliferation/apoptosis of cancer cells^40^. Since two selective ROCK inhibitors, Y-27632 and Fasudil, reportedly increase sphere formation and SOX2 expression by a glioblastoma cell line (U87-MG) and patient-derived glioblastoma xenoline (JX12) (ref. 41), Rho signaling downstream of p190 RhoGAP may be involved in PTPRZ-mediated regulation of glioma stem cell function.

Another R5 RPTP subfamily member, PTPRG, has previously been reported to be overexpressed in most high-grade astrocytomas^19^. However, PTPRG has been postulated as a tumor suppressor in breast cancer cells because its expression is decreased in breast tumor tissues, and its overexpression inhibits anchorage-independent growth and proliferation of breast cancer cells^41^. These findings suggest different roles for PTPRG in different neoplastic cell types. We previously demonstrated that the knockdown of *Ptprz*, but not *Ptprg*, inhibits the migration ability of C6 glioblastoma cells expressing both PTPRZ and PTPRG (ref. 6). To the best of our knowledge, no studies have yet addressed the functional roles of PTPRG in cancer stem cells. We herein reveal that the knockdown of *Ptprg* also inhibits sphere formation and *Sox2* expression in C6 cells (Fig. 9). Therefore, the simultaneous inhibition of the two R5 RPTP subfamily members may be more effective than single inhibition toward achieving an anti-glioma effect.

**Figure 11:**
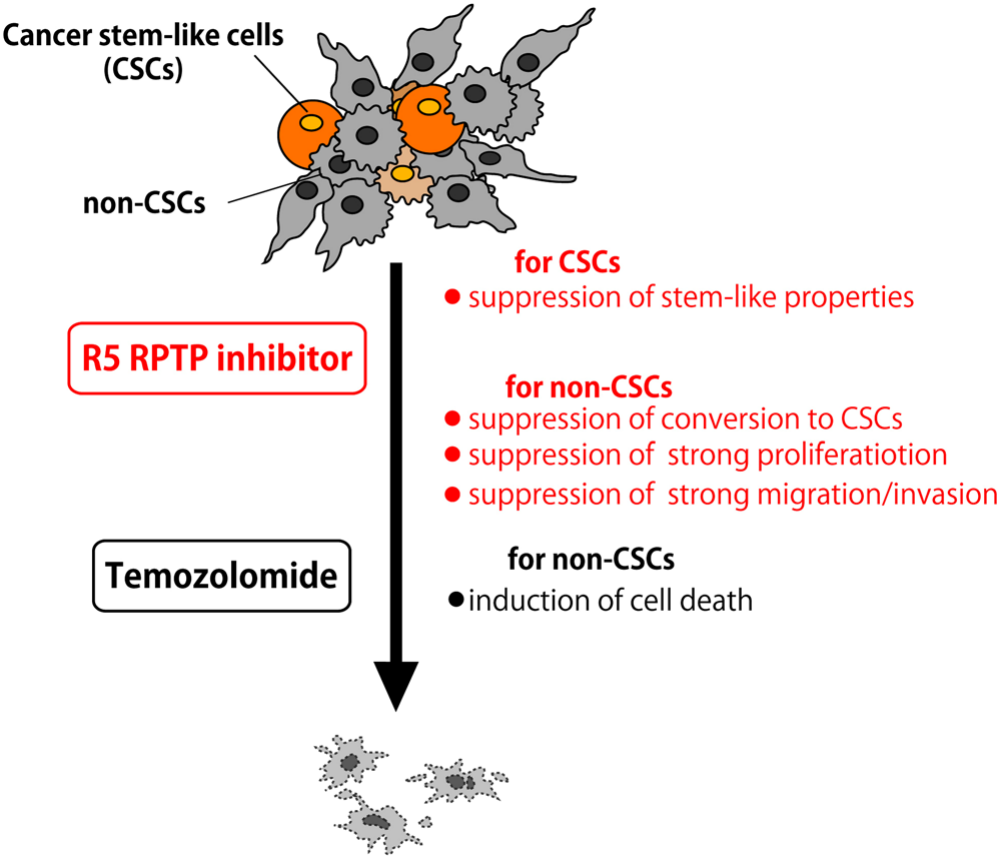
CSC differentiation effects of the pharmacological inhibition by R5 RPTPs. Because cancer stem-like cells (CSCs) contribute to therapeutic resistance and tumor recurrence in malignant tumors including glioblastoma^24^, a more effective therapy should target chemoresistant CSCs and proliferating and invading cancer cells. PTPRZ is strongly expressed in malignant gliomas^20, 21^, and the expression levels of PTPRZ transcripts are closely associated with cancer stemness in primary human glioblastomas^26^. The short receptor form, PTPRZ-B, is the major PTPRZ isoform in rat C6 and human U251 glioblastoma cells (Fig. 1D), the expression of which is significantly stronger in sphere-forming cells than normal cultured cells. The cell-permeable R5 RPTP inhibitor, NAZ2329, suppresses the stem cell-like properties of CSCs and the strong proliferation and migration characteristics of adherent glioblastoma cells (non-CSCs). The synergistic antitumor effects of NAZ2329 with temozolomide (TMZ) on the C6 glioblastoma xenograft, which was previously reported to be relatively resistant to TMZ (ref. 34), indicates that the combined use of R5 RPTP inhibitors and TMZ will be an effective treatment for malignant gliomas.

In summary, compelling evidence has suggested that a rare subpopulation of tumor cells known as CSCs contributes to tumor initiation, tumor progression, therapeutic resistance, and tumor recurrence in malignant tumors including glioblastoma^24^. Many therapeutic approaches have been devised to target CSCs but with limited success^42^. To overcome this issue, future therapy should target the chemoresistant CSCs more effectively, with the aim of inducing the conversion of CSCs to non-CSCs, or suppressing the spontaneous conversion of non-CSCs to CSCs and/or the self-renewal ability of CSCs. The alkylating agent TMZ is the most effective drug for the treatment of glioblastoma. However, long-term therapy results in the occurrence of drug-resistant glioblastoma cells^24^. Our combined treatment of TMZ with NAZ2329 significantly delayed tumor growth relative to the single treatments with either TMZ or NAZ2329 (Fig. 10), suggesting that R5 RPTP inhibitors enhanced the sensitivity of malignant gliomas to the alkylating agent (Fig. 11). Together with the previous report that identified PTPRZ1 as a stemness classifier gene by single-cell RNA sequencing of primary human glioblastomas^26^, our results indicate that the R5 RPTP subfamily comprises a novel group of molecular targets for differentiation-inducing therapy for malignant gliomas.

## Methods

### Ethics statement and experimental animals

All procedures in this study were approved by the Institutional Animal Care and Use Committee of the National Institutes of Natural Sciences, Japan; approval numbers are 15A026 and 16A146, and were performed in accordance with the guidelines of the institutional committee for the use of animals for research. All surgeries were performed under isoflurane anesthesia and all efforts were made to minimize suffering. BALB/c-*nu*/*nu* mice (females, 4 weeks old) were purchased from Charles River Japan.

### Enzymes and antibodies

Recombinant proteins corresponding to the entire intracellular regions (ICRs) of PTPRZ1, PTPRA, and PTPRM were expressed using a baculovirus-silkworm expression system, and purified as described^6^. The ICRs of PTPRG, PTPRS, and PTPRB and the catalytic domains of PTPN1 and PTPN6 were expressed as glutathione-S-transferase (GST) fusion proteins from each pGEX plasmid in *Escherichia coli* strain BL21 (ref. 6). GST fusion proteins were purified by glutathione affinity chromatography as described^43^. Chondroitinase ABC (chABC) was purchased from Sigma-Aldrich (catalog #C3667). Anti-PTPRZ-S, rabbit polyclonal antibodies against the extracellular region of PTPRZ was described previously (ref. 44). The following are the specificities and sources of the commercially available antibodies used in the present study: Anti-RPTPβ (a monoclonal antibody against the intracellular domain of PTPRZ1 receptors, #610179, BD Biosciences), anti-SOX2 (#ab97959, Abcam), anti-POU3F2 (#12137, Cell Signaling), anti-OLIG2 (#AB9610, Millipore), anti-SALL2 (#12679–1-AP, Proteintech Group), anti-GAPDH (#ab9482, Abcam) anti-phosphotyrosine (PY20; #ab16389, Abcam), and anti-pY118-paxillin (#2541, Cell Signaling), mouse anti-paxillin antibody (#610569, BD Bioscience), anti-MBP (#sc-13914, Santa Cruz Biotechnology), and anti-NG2 proteoglycan (#AB5320, Millipore).

### Chemical Synthesis

The synthesis of NAZ2329 and its spectral data were provided as a supplementary file.

### Structural elucidation of human PTPRZ1-D1 complexed with NAZ2329

Crystals of human PTPRZ1-D1 (amino acid residues, 1,698-2,000) obtained as previously described^6^ were placed in crystallization reservoir solution with 1 mM NAZ2329 overnight. The crystal structure determination of PTPRZ1-D1 complexed with NAZ2329 was performed as described^6^. Interactions between PTPRZ and NAZ2329 were identified using LigPlot^+^ (ref. 45).

### Expression plasmids

The mammalian expression plasmid of human PTPRZ1-B (pZeo-hPTPRZ1-B) was described previously^6^, and that of V1911F PTPRZ1-B mutant was generated using pZeo-hPTPRZ1-B as template with a Quikchange multisite-directed mutagenesis kit (Stratagene). Point mutants of the PTPRZ1-ICR, PTPRG-ICR, and PTPN1 catalytic domain were generated from pGEX-6P-PTPRZ1-ICR, pGEX-6P-PTRG-ICR, and pGEX-6P-PTPN1 (ref. 6), respectively, using a Quikchange multisite-directed mutagenesis kit (Stratagene).

### *In vitro* PTPase assays

For determination of IC_50_ values, Lineweaver-Burk plot, and inhibitor sensitivity assays, recombinant PTP proteins were preincubated with a dilution series of the compound in assay buffer (100 mM acetate, 50 mM Tris, and 50 mM Bis-Tris, pH 6.5 containing 100 μg/ml BSA, 5 mM DTT, and 0.01% Brij-35) for 1 h, and the enzyme-inhibitor mixture was added to an equal volume of 40 μM DiFMUP (6,8-difluoro-4-methylumbiliferyl phosphate, #D6567, Thermo Fisher Scientific) solution to initiate the reaction. The hydrolysis of DiFMUP was continuously monitored as an increase in fluorescence at 455 nm (excitation at 358 nm) for ~100 s using a spectrofluorometer (FI-4500, Hitachi) at room temperature. The slope of the fluorescence signal was defined as catalytic activity. IC_50_ values were calculated by the conventional linear interpolation method. We examined preincubation effect, reversibility, and time dependence of PTP inhibition by NAZ2329 as described in the figure legends.

### Glioblastoma cell culture and electroporation

Rat C6 glioblastoma cells that have been maintained in our laboratory were used. *RZ*-KD#2, a C6 clone that is stably transfected with an shRNA expression vector targeting *Ptprz*, was described previously^6^. Human U251 glioblastoma cells were purchased from the American Type Culture Collection (ATCC). C6 and U251 cells were maintained in Dulbecco’s modified Eagle’s medium (DMEM, #11995-040, Thermo Fisher Scientific) supplemented with 10% fetal bovine serum (FBS, Nichirei Bioscience) and 100 U/ml penicillin–streptomycin (#15140122, Thermo Fisher Scientific) in a humidified incubator at 37 °C with 5% CO_2_.

Cells (2 × 10^6^ cells) were electroporated with 100 pmol siRNA (for rat *Ptprz*, siRNA ID; SASI_Rn01_00053281: for rat *Ptprg* siRNA, siRNA ID; SASI_Rn01_00066571: Sigma-Aldrich) or control siRNA using Amaxa Nucleofector (Amaxa), or 4 µg expression plasmid according to the manufacturer’s protocol. Twenty-four to 36 h after electroporation, cells were used for experiments as described previously (ref. 6). Predesigned human *PTPRZ1* shRNA plasmid DNA (TRC number: TRCN00000356375) was purchased from Sigma-Aldrich. After the electroporation, cells were selected by limited dilution in the presence of 5 µg/ml of puromycin, and obtained stably *PTPRZ1*-knockdown cell lines including *RZ1*-KD#5U.

### Boyden chamber assay

This assay was performed as described previously^6^. Briefly, cells were preincubated with compounds for 30 min, and then transferred onto a laminin-coated transwell insert in which the lower chambers contained epidermal growth factor (EGF, #E9644, Sigma-Aldrich). Cells were allowed to migrate for 3 h, and the number of cells that migrated to the lower surface was counted under a conventional fluorescence microscope.

### Cell proliferation assay

Cells (3 × 10^4^ cells) were inoculated into a 24-well plastic tissue culture plate with 500 µl of DMEM supplemented with 2% FBS and the indicated compounds for 48 h. Cells were harvested by trypsinization and then manually counted with a hemacytometer.

### Sphere formation assay

Cells were inoculated at 5 × 10^3^ cells per 96-well uncoated polystyrene plate (# 9018, Corning) or 5 × 10^4^ cells per 35-mm plastic petri dish (#351008, Corning), and cultured in a serum-free medium containing DMEM/F12 with 1x B27 supplement (#17504044, Thermo Fisher Scientific), EGF (20 ng/ml), and basic fibroblast growth factor (bFGF, 20 ng/ml, #064-05381, Wako pure chemical). After a 7-day culture, cells were fixed with 10% neutral formalin and pictured under a conventional microscope. The number of spheroids of approximately 70 µm in diameter were counted manually using Adobe Photoshop CS6 (Adobe).

### cDNA synthesis and quantitative real-time PCR

cDNA synthesis and quantitative real-time PCR. Total RNA of cultured cells was isolated with TRIzol Reagent kit (#12183555, Thermo Fisher Scientific). cDNAs were synthesized using the PrimeScript RT reagent Kit with gDNA Eraser (Takara Bio), and used as a template for real-time PCR using a commercial kit (TaKaRa One Step SYBR, Takara Bio) on a real-time PCR system (StepOnePlus Real Time PCR System, Thermo Fisher Scientific). The relative mRNA expression of rat *Ptprz*, *Ptprg*, and *Sox2* was normalized to that of glyceraldehyde-3-phosphate dehydrogenase (*Gapdh*). Sequences of commercial primer sets (perfect real-time primer support system, Takara-Bio) are as follows: *Ptprz* (NM_013080.2, 6889–7023), forward 5′-atgaggccgggagtcttcac-3′ and reverse 5′-tccatcaggcaaagctgcac-3′; *Ptprg* (NM_134356.1, 3356–3445), forward 5′-agcatatcaggacacagcggaac-3′ and reverse 5′-tcccgagaatggcttccaac-3′; *Sox2* (NM_001109181.1, 973–1054), forward 5′-gtcagcgccctgcagtacaa-3′ and reverse 5′-gcgagtaggacatgctgtaggtg-3′, or *Gapdh* (NM_017008.4, 241–383) forward 5′-ggcacagtcaaggctgagaatg-3′ and reverse 5′-atggtggtgaagacgccagta-3′. Relative quantities of the target mRNAs were normalized to GAPDH.

### Protein extraction and chABC digestion

Proteins were extracted from cultured cells with 1% Nonidet P-40 in 10 mM Tris-HCl, pH 7.4, 150 mM NaCl (TBS) containing 1 mM vanadate, 10 mM NaF, and protease inhibitors (EDTA-free complete, #11873580001, Roche Molecular Biochemicals). To detect PTPRZ proteins by Western blotting, extracts were subjected to chABC digestion and 10-µl aliquots were mixed with an equal volume of 0.2 M Tris-HCl, 60 mM sodium acetate, and 10 mM EDTA, pH 7.5 containing 250 micro-units of chABC or not (control) at 37 °C for 1 h.

### Immunoprecipitation and Western blotting assays

After precleaning the extracts with Protein G Sepharose (#17-0618-02, GE Healthcare), samples were subjected to immunoprecipitation with Protein G Sepharose coated with a mouse anti-paxillin antibody. Samples were mixed with an equal volume of 2 × SDS-PAGE sample buffer (containing 200 mM dithiothreitol), boiled for 5 min, and then separated on a 5–20% gradient polyacrylamide gel (#E-R520L, Atto Corp.). Proteins were transferred to a polyvinylidene difluoride membrane (Immobilon-P, Millipore) for 1 h using a conventional semidry electrotransfer (1.3 mA per cm^2^). The membrane was incubated for 1 h in a blocking solution (For protein detections, 4% nonfat dry milk and 0.1% Tween 20 in TBS, or for pY118-paxillin detection and Tyr-phosphorylation, 1% BSA and 0.1% Tween 20 in TBS) and incubated overnight with indicated primary antibodies, followed by incubation with horseradish peroxidase (HRP)–conjugated secondary antibodies (for rabbit IgG, #RPN4301; for mouse IgG, RPN4201V; GE Healthcare). The binding of these antibodies was detected with Luminata Forte Western HRP substrate (Millipore) and imaged with a chemiluminescent image analyzer system (EZ-Caputure MG, Atto Corp).

### OL1 cell culture and Immunocytofluorescence staining

The preparation of mouse oligodendrocyte-lineage OL1 cells was described previously^15^. In the differentiation assay, OL1 cells (2.0 × 10^4^ cells) were cultured on a poly-L-ornithine (#P3655, Sigma-Aldrich)-coated 35-mm plastic dish in Knock-out DMEM/F-12 (#21331-020, Thermo Fisher Scientific) supplemented with 1 × GlutaMAX supplement (#35050061, Thermo Fisher Scientific), 1 × StemPro neural supplement (#A1050801, Thermo Fisher Scientific), 10 μg/ml PDGF-AA (#165-25541, Wako Pure Chemical), 10 nM biotin (#B4501, Sigma-Aldrich), and 30 ng/ml thyronine (#T2752, Sigma-Aldrich), and 30 ng/ml thyroxine (#T2376, Sigma-Aldrich). On the 10th day of the culture, differentiation from oligodendrocyte precursor cells to oligodendrocytes was assessed was described previously^14–16^. Briefly, cells were fixed and stained with anti-NG2 (a specific marker for oligodendrocyte precursor cells) and anti-MBP (a marker for matured oligodendrocytes), and the observed with a standard fluorescence microscope (Biozero BZ8000, Keyence). Differentiation from oligodendrocyte precursor cells to oligodendrocytes was expressed as the ratio of MBP-positive cells to NG2-positive cells.

### Xenograft tumor mouse model and drug treatment

Parental or *Ptprz*-knockdown C6 cells (5 × 10^6^ cells) suspended in a volume of 100 µl were subcutaneously injected into the left hind limbs of BALB/c-*nu*/*nu* mice (females, 4 weeks old mice were purchased from Japanese Charles River Co. Ltd.). Tumor growth was measured with calipers and calculated by the formula: volume = length × width × width × 0.5, as previously described^46^. The drug treatment initiated when the tumor size reached 150 mm^3^. Mice were randomly divided into four treatment groups: DMSO control, NAZ2329, temozolomide, and the combination of NAZ2329 with temozolomide. All mice were euthanized at a humane endpoint (>3,000 mm^3^ tumor size, 40 days after the drug treatment, or massive tumor necrosis).

### Image and statistical analyses

Quantitative image analyses were performed using ImageJ (NIH) or Adobe Photoshop CS6 (Adobe Systems). Significance was established using the Student’s *t*-test or an analysis of variance (ANOVA) and *post-hoc* test (Bonferroni) (SPSS software version 20.0, IBM).

### Data availability statement

All data generated or analyzed during this study are included in this published article and its Supplementary Information files.

## Electronic supplementary material

Supplementary Information
