# Supplementary material for: Targeting PTPRZ inhibits stem cell-like properties and tumorigenicity in glioblastoma cells
Source: Sci Rep. 2017 Jul 17;7:5609. doi: 10.1038/s41598-017-05931-8 (PMC5514153; doi:10.1038/s41598-017-05931-8)
Supplement: Supplementary file 1 — Supplementary Information [file 41598_2017_5931_MOESM1_ESM.pdf]

Supplementary Information

Targeting PTPRZ inhibits stem cell-like properties and tumorigenicity in glioblastoma cells

Akihiro Fujikawa, Hajime Sugawara, Taisaku Tanaka, Masahito Matsumoto, Kazuya Kuboyama, Ryoko Suzuki, Naomi Tanga, Atsuto Ogata, Makoto Masumura, and Masaharu Noda

Synthesis of NAZ2329. The synthesis of NAZ2329 was performed stepwise as shown below (see supplementary Figs. S5). ^{1}H NMR spectra were obtained on a Brucker Avance III (400 MHz) spectrometer in the indicated solvent. Chemical shifts and coupling constants in ^{1}H NMR signals were elucidated using the analyzing software, ACD Spectrus Processor (2012 Release). Electrospray ionization mass spectra were obtained on an Agilent G1956A MSD spectrometer system. Chemical reagents and solvents of the highest grade were purchased from Sigma-Aldrich Co. LLC., Tokyo Chemical Industry Co., Ltd., Wako Pure Chemical Industries Ltd., Kanto Chemical Co., Inc., or Nacalai Tesque, and used without purification. Flash column chromatography was performed using Purif-Pack SI 30 μm supplied by Shoko Scientific.

3-{[2-Methoxy-5-(trifluoromethyl)benzyl]thio}thiophene-2-carboxylic acid (compound 3): A 28% solution of sodium methoxide in methanol (7.11 ml) and 2-(bromomethyl)-1-methoxy-4-(trifluoromethyl)benzene (compound **2**, 4.96 g) were added to a solution of 3-mercaptothiophene-2-carboxylic acid (compound **1**, 2.95 g) in dimethyl sulfoxide (100 ml), and the mixture was stirred at room temperature for 1 h. In the next step, 1.0 M hydrochloric acid and ethyl acetate were added to the reaction mixture. The precipitate that appeared was collected by filtration to give compound **3** (2.50 g, 38.9%). The filtrate was extracted with ethyl acetate. The organic layer was washed with brine, dried over anhydrous sodium sulfate, and concentrated *in vacuo*. The residue was purified on silica gel column

chromatography employing a gradient of 97:3 to 90:10 with chloroform/methanol to give compound 3 (0.98 g, 15.3%). ^{1}H NMR (DMSO-*d6*) δ: 7.86 (1H, d, *J* = 5.14 Hz), 7.73 (1H, d, *J* = 2.13 Hz), 7.65 (1H, dd, *J* = 8.66, 2.10 Hz), 7.26 (1H, d, *J* = 5.10 Hz), 7.22 (1H, d, *J* = 8.66 Hz), 4.31 (2H, s), 3.92 (3H, s); MS (ESI): 349 $(M+H)^+$.

Methyl 3-{[2-methoxy-5-(trifluoromethyl)benzyl]thio}thiophene-2-carboxylate (compound 4): Thionyl chloride (1.46 ml) was added to a solution of compound **3** (3.48 g) in methanol (50 ml) at 0 °C, and the mixture was stirred at reflux for 2 days. The mixture was then concentrated. Water and ethyl acetate were added to the residue, and the resulting biphasic solution was separated. The organic layer was washed with brine, dried over anhydrous sodium sulfate, and concentrated *in vacuo*. The residue was purified on silica gel column chromatography employing a gradient of 95:5 to 80:20 with hexane/ethyl acetate to give compound **4** (3.08 g, 85.0%). ^{1}H NMR ($CDCl_3$) δ: 7.63 (1H, d, *J* = 2.01 Hz), 7.52 (1H, dd, *J* = 8.53, 2.00 Hz), 7.47 (1H, d, *J* = 5.27 Hz), 7.00 (1H, d, *J* = 5.27 Hz), 6.94 (1H, d, *J* = 8.50 Hz), 4.28 (2H, s), 3.92 (3H, s), 3.87 (3H, s); MS (ESI): 363 $(M+H)^+$.

Methyl 3-{[2-hydroxy-5-(trifluoromethyl)benzyl]thio}thiophene-2-carboxylate (compound 5): A 17% solution of boron tribromide in dichloromethane (21 ml) was added to a solution of compound **4** (3.0 g) in dichloromethane (45 ml) at 0 °C, and the mixture was stirred at 0 °C for 3 h. Methanol was added to the mixture at 0 °C, and the resulting mixture was then stirred at room temperature for 15 min. The mixture was concentrated, water and ethyl acetate were added to the residue, and the resulting biphasic solution was separated. The organic layer was washed with saturated sodium hydrogen carbonate aqueous solution and brine, dried over anhydrous sodium sulfate, and concentrated *in vacuo*. The residue was purified on silica gel column chromatography employing a gradient of 75:25 to 60:40 with hexane/ethyl acetate to give compound **5** (0.87 g, 30%). ^{1}H NMR ($CDCl_3$) δ: 7.51 (1H, d, *J* = 5.14 Hz), 7.49 (1H, d, J = 1.90 Hz), 7.45 (1H, dd, *J* = 8.47, 1.94 Hz), 7.13 (1H, d, *J* = 5.10 Hz), 6.97 (1H, d, *J* = 8.50 Hz), 4.26 (2H, s), 3.92 (3H, s); MS (ESI): 349 $(M+H)^+$.

3-{[2-Ethoxy-5-(trifluoromethyl)benzyl]thio}thiophene-2-carboxylic acid (compound 6): A 60% dispersion of sodium hydride in mineral oil (0.24 g) was added to a solution of compound **5** (0.87 g) in *N*,*N*-dimethylformamide (20 ml) at 0 °C, and the mixture was stirred at room temperature for 5 min.

Ethyl iodide (0.61 ml) was then added to the mixture 0 °C, and the resulting mixture was stirred at room temperature for 3 h. Water was added to the mixture, and the resulting mixture was extracted with ethyl acetate. The organic layer was washed with brine, dried over anhydrous sodium sulfate, and concentrated *in vacuo* to give a crude product (0.98 g).

A 4.0 M sodium hydroxide aqueous solution (3.0 ml) was added too a solution of the obtained compound (0.90 g) in methanol (30 ml), and the mixture was stirred at reflux for 3 h. The mixture was concentrated, and water and ethyl acetate were added to the residue, and the resulting biphasic solution was then separated. The organic layer was washed with 1.0 M hydrochloric acid and brine, dried over anhydrous sodium sulfate, and concentrated *in vacuo*. The residue was purified on silica gel column chromatography employing a gradient of 99:1 to 90:10 with chloroform/methanol to give compound **6** (0.33 g, 36%). ^{1}H NMR (CD_3OD) δ: 7.61-7.64 (2H, m), 7.52 (1H, dd, J = 8.60, 1.82 Hz), 7.07-7.13 (2H, m), 4.31 (2H, s), 4.18 (2H, q, J = 7.03 Hz), 1.45 (3H, t, J = 7.03 Hz); MS (ESI): 363 $(M+H)^+$.

3-{[2-Ethoxy-5-(trifluoromethyl)benzyl]thio}-*N*-(phenylsulfonyl)thiophene-2-carboxamide (NAZ2329): A 60% dispersion of sodium hydride in mineral oil (40 mg) was added to a solution of benzenesulfonamide (0.16 g) in *N*,*N*-dimethylformamide (10 ml) at 0 °C, and the mixture was stirred at room temperature for 15 min. The mixture was added to a solution, which was prepared by stirring compound **6** (0.30 g) and 1,1'-carbonyldiimidazole (0.16 g) in *N*,*N*-dimethylformamide (10 ml) at room temperature, at 0 °C. The resulting mixture was stirred at room temperature for 2 days and at 50 °C for 5 h. Then, 1.0 M hydrochloric acid was added to the reaction mixture, and the resulting mixture was extracted with ethyl acetate. The organic layer was washed with brine, dried over anhydrous sodium sulfate, and concentrated *in vacuo*. The residue was purified on silica gel column chromatography employing a gradient of 99:1 to 90:10 with chloroform/methanol to give NAZ2329 (0.28 g, 67%). ^{1}H NMR (CD_3OD) δ: 7.95-8.02 (2H, m), 7.89 (1H, s), 7.75 (1H, d, J = 5.14 Hz), 7.63-7.70 (1H, m), 7.53-7.61 (2H, m), 7.46-7.52 (1H, m), 7.21 (1H, d, J = 5.14 Hz), 7.11-7.14 (1H, m), 7.11-7.14 (1H, m), 7.08 (1H, d, J = 8.53 Hz), 4.14-4.23 (4H, m), 1.44 (3H, t, J = 6.96 Hz); MS (ESI): 502 $(M+H)^+$.

Legends to Supplementary Figures

Supplementary Figure S1. Aberrant expressions of core transcription factors in *Ptprz*-knockdown glioblastoma cells. (**A**) Western using antibodies against SOX2, OLIG2, POU3F2, and SALL2. Parental C6 and *RZ*-KD#2, and parental U251 and *RZ1*-KD#5U cells were cultured in the serum-supplemented normal medium. SALL2 proteins were not detected in C6 or U251 cells. Amounts of sample loading were verified by immunostaining with GAPDH. Images are representative of five independent cultures. The plots show the arbitrary densitometric units of the staining intensity in *PTPRZ*-knockdown cells relative to those of the parental cells. *, $p < 0.05$; **, $p < 0.01$ (Student's *t*-test). (**B**) Full-length blots of those present in A are shown.

Supplementary Figure S2. *In vitro* PTP assays. Concentration-inhibition curves of NAZ2329 and temozolomide for the whole intracellular region (D1+D2) and the D1 domain (D1) of PTPRZ1. The curve of PTPRZ1 (D1+D2) is the same one shown in Fig. 4F. Assays were performed using DiFMUP. IC_{50} values are indicated in the inset. Data are averaged from two separate experiments. No significant inhibitions were observed with temozolomide.

Supplementary Figure S3. Identification of an allosteric site for NAZ2329. (**A**) Sequence alignment of the α3 helix in D1 and D2 domains in human PTP domains. Total 37 human members of the classical PTP family are classified into eight receptor-type transmembrane subtypes (R1/6 to R7) and nine non-transmembrane subtypes (NT1 to NT9): Total 49 PTP domains (37 PTP-D1s and 12 PTP-D2s) are shown. In tandem-domain RPTPs, the membrane proximal D1 domain displays catalytic activity while the distal D2 domain is either inactive or has negligible (RA and RE) catalytic activity[10]. Val and Phe residues at positions corresponding to Val-1911 of PTPRZ1 are colored in blue and red, respectively. (**B**) Sensitivity of point substitution mutants of PTPs to SCB4380 (10 μM) or vanadate (200 μM). Data were analyzed as in Fig. 4D. *, $P < 0.05$; **, $P < 0.01$, significantly different from the wild-type enzyme (Student's *t*-test). The Val to Phe mutation of PTPRZ1 and PTPRG hardly affected their sensitivity to SCB4380 or vanadate.

The Phe to Val mutation of PTPN1 decreased its vanadate sensitivity, which was inverse to that for NAZ2329.

Supplementary Figure S4. Effects of the NAZ2329 treatment on human U251 glioblastoma cells. Effects of NAZ2329 on the proliferation (*A*), migration (*B*), and SOX2 expression (*C*) of human U251 cells. Experiments were performed as in Figure 3. Images are representative of five independent culture conditions. Scale bars, 100 μm. *, $P < 0.05$; **, $P < 0.01$, significantly different from the vehicle (one-way ANOVA with Bonferroni *post hoc* tests).

Supplementary Figure S5. Scheme of NAZ2329 synthesis. Reagents and conditions: (a) NaOMe in MeOH, DMSO, room temperature (rt); (b) thionyl chloride, MeOH, reflux; (c) BBr_3, CH_2Cl_2, 0 °C; (d) NaH, EtI, DMF, rt; (e) aq. NaOH, MeOH, reflux; (f) benzenesulfonamide, NaH, CDI, DMF, rt and then 50 °C.

Supplementary Figure S6. Full-length blot and gel images of those in Figs. 1 and 2.

Supplementary Figure S7. Full-length blot and gel images of those in Fig. 7.

Supplementary Figure S8. Full-length blot and representative microscopic images of those in Figs. 8A to 8D.

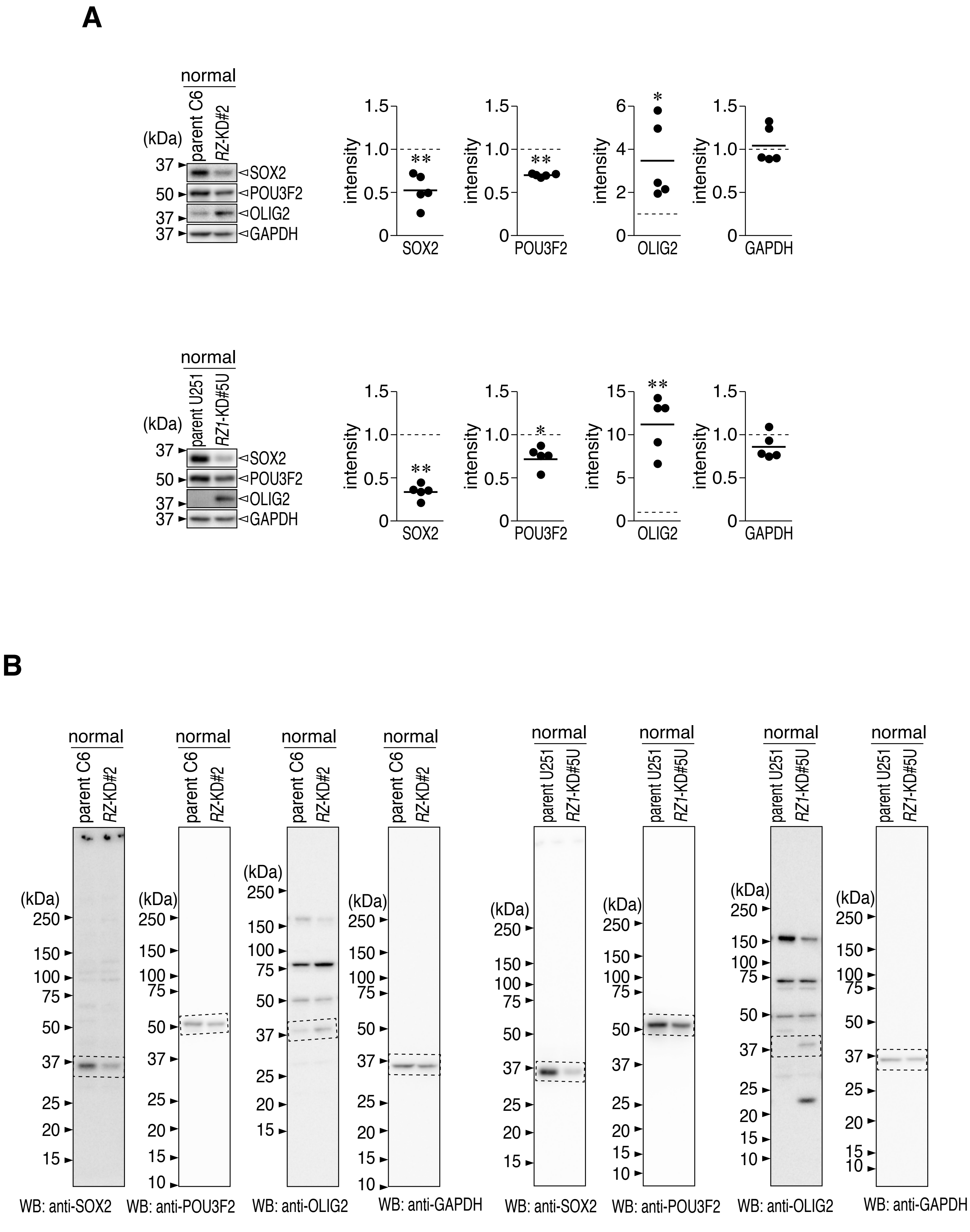

Supplementary Figure S1

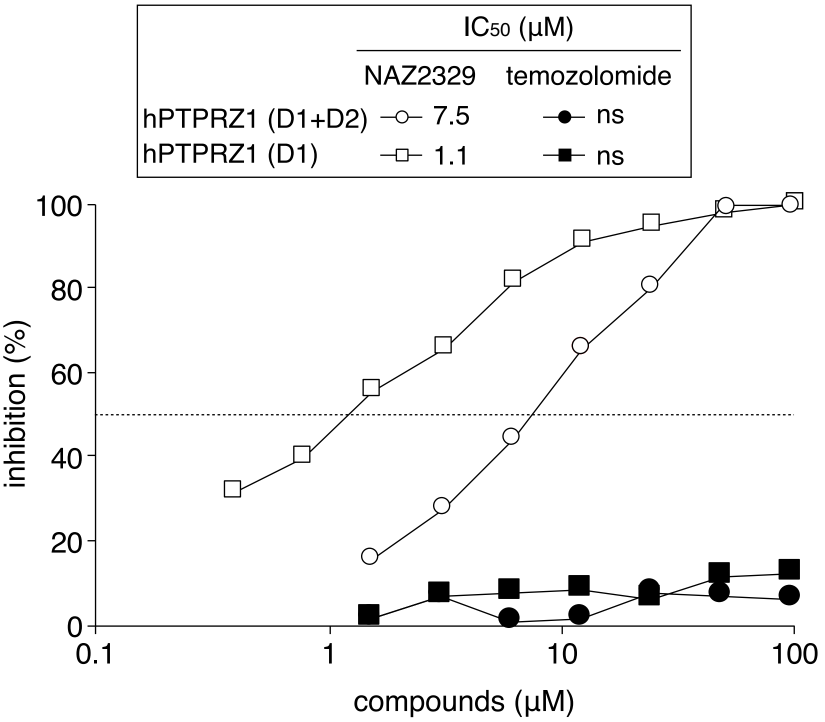

Supplementary Figure S2

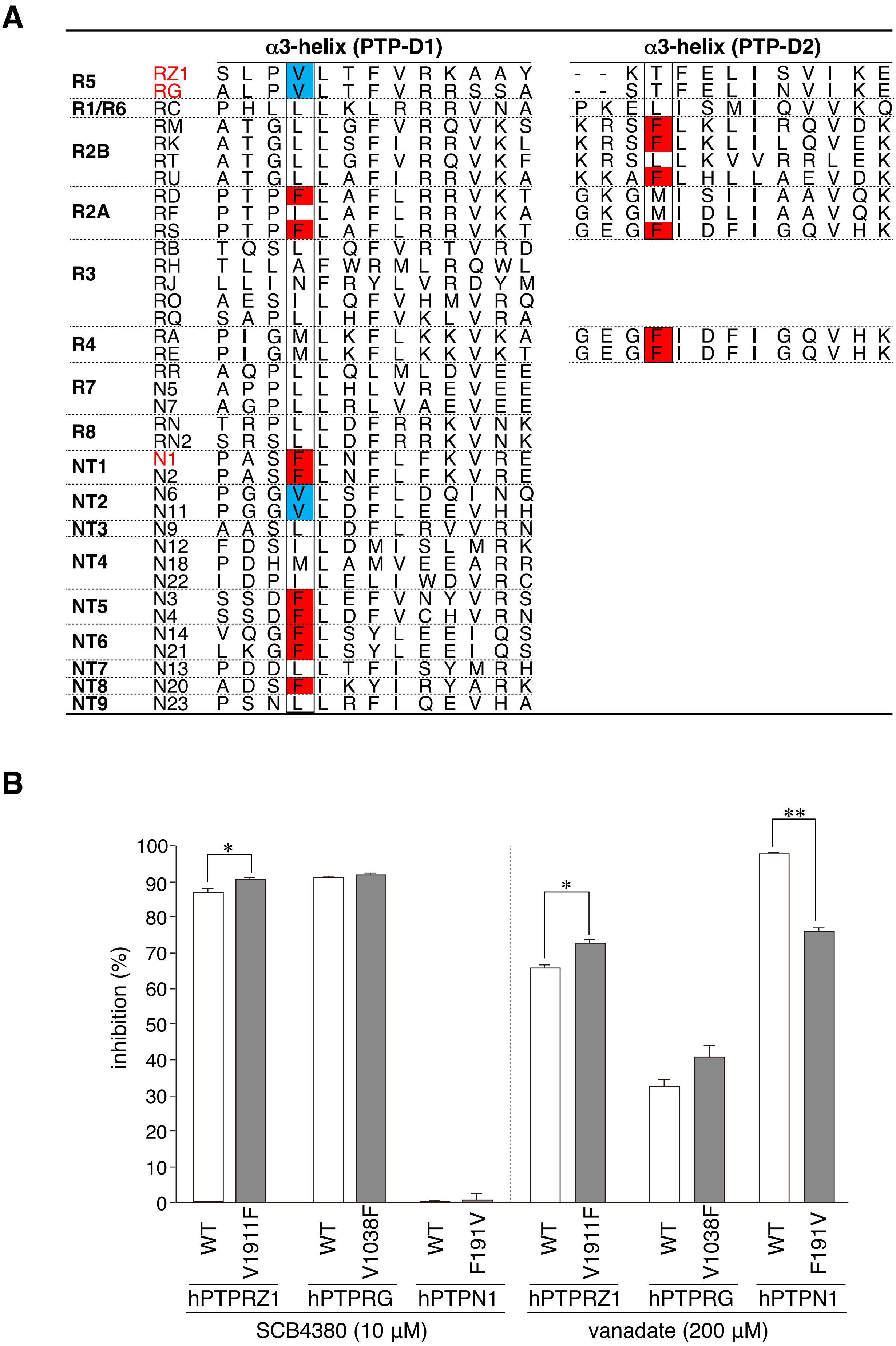

Supplementary Figure S3

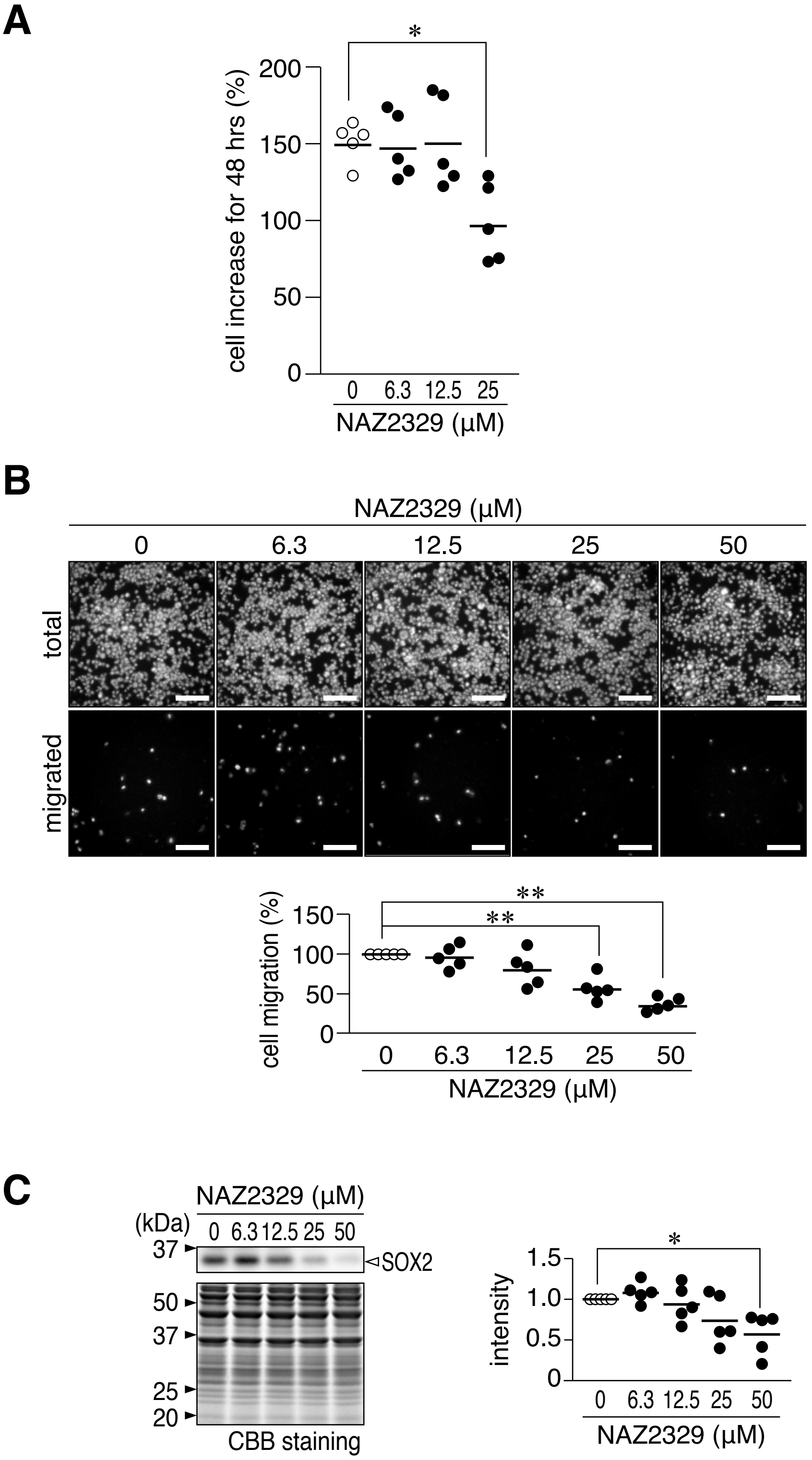

Supplementary Figure S4

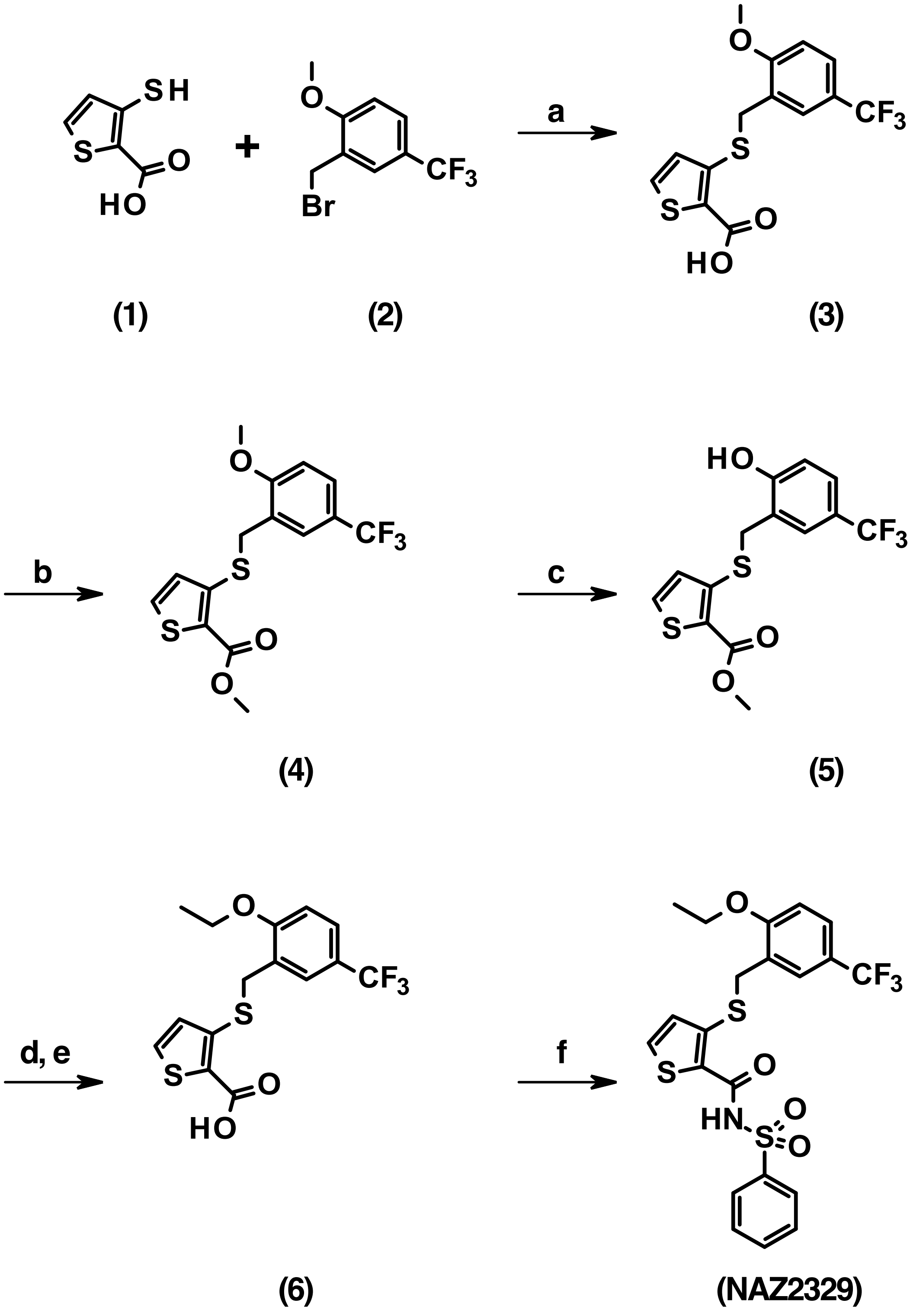

Supplementary Figure S5

For Fig. 1

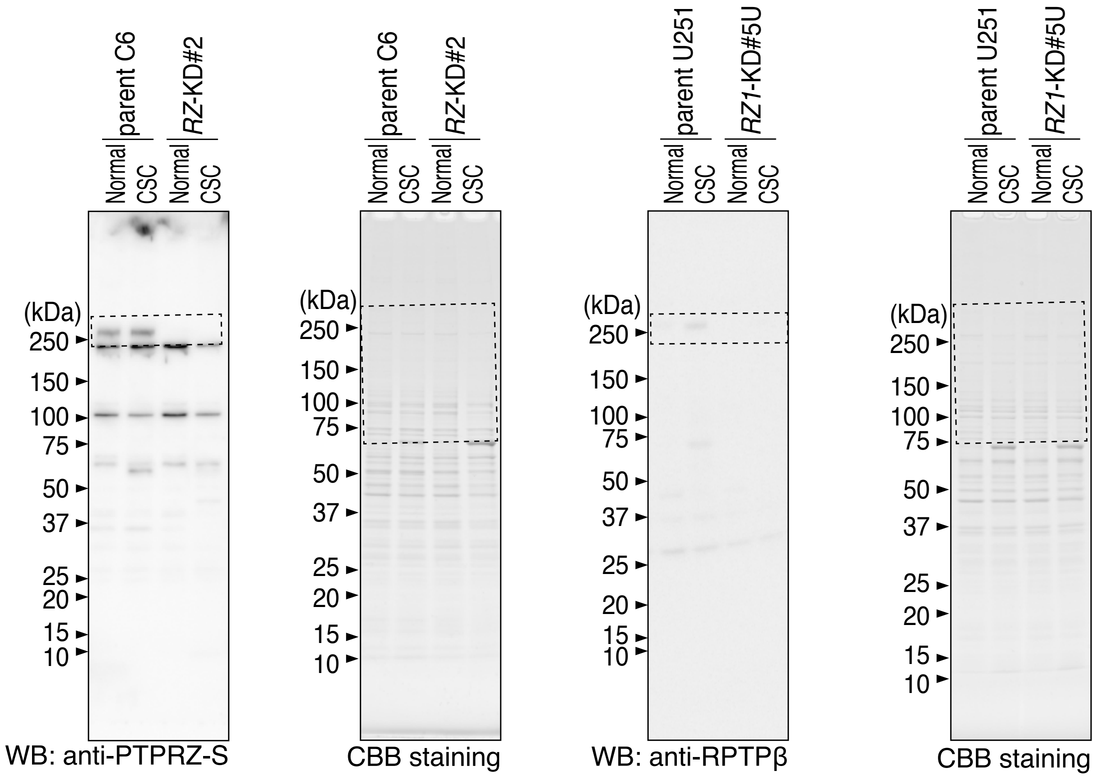

For Fig. 2

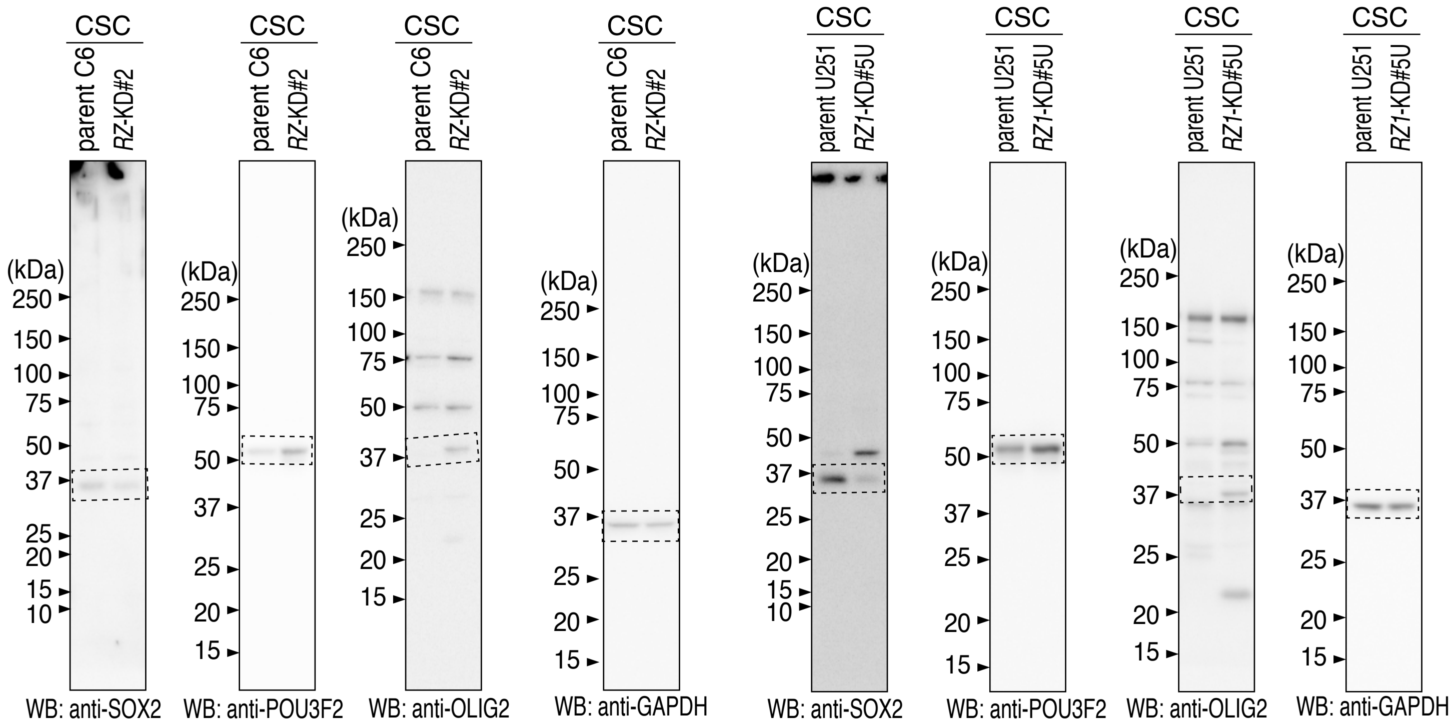

Supplementary Figure S6

For Fig. 7A　　　　**For Fig. 7E**

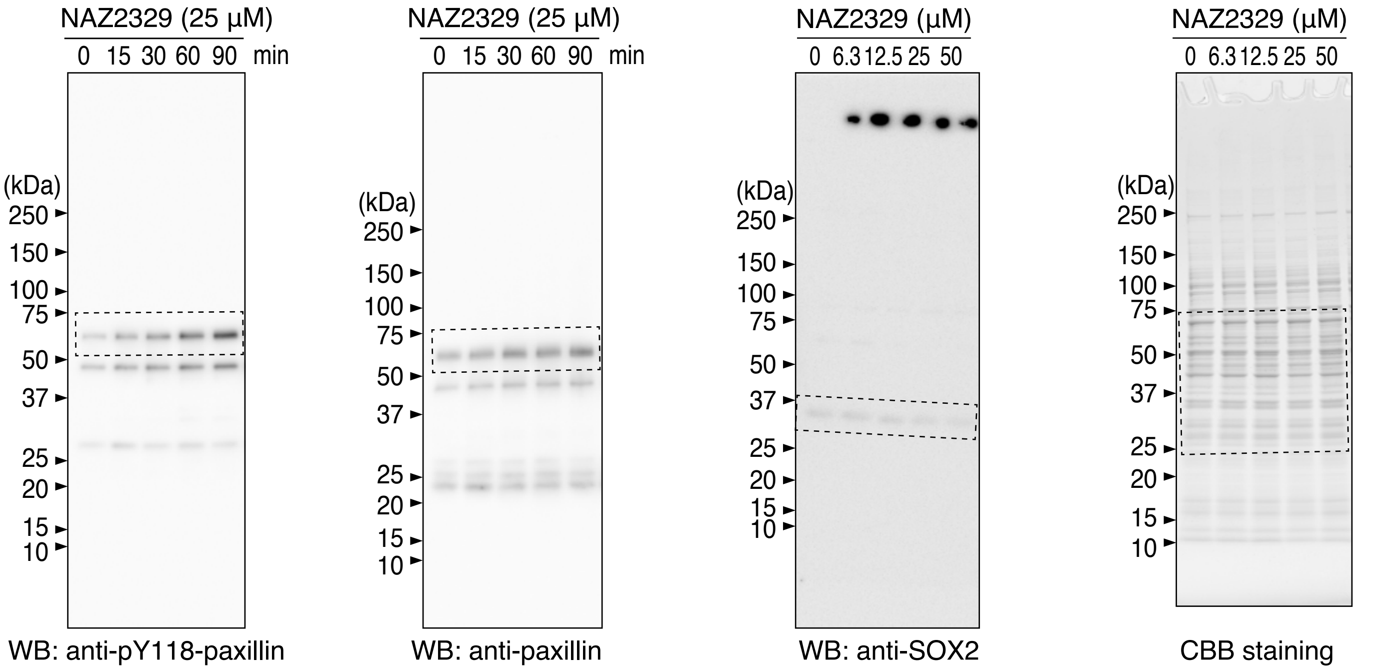

Supplementary Figure S7

For Fig. 8A

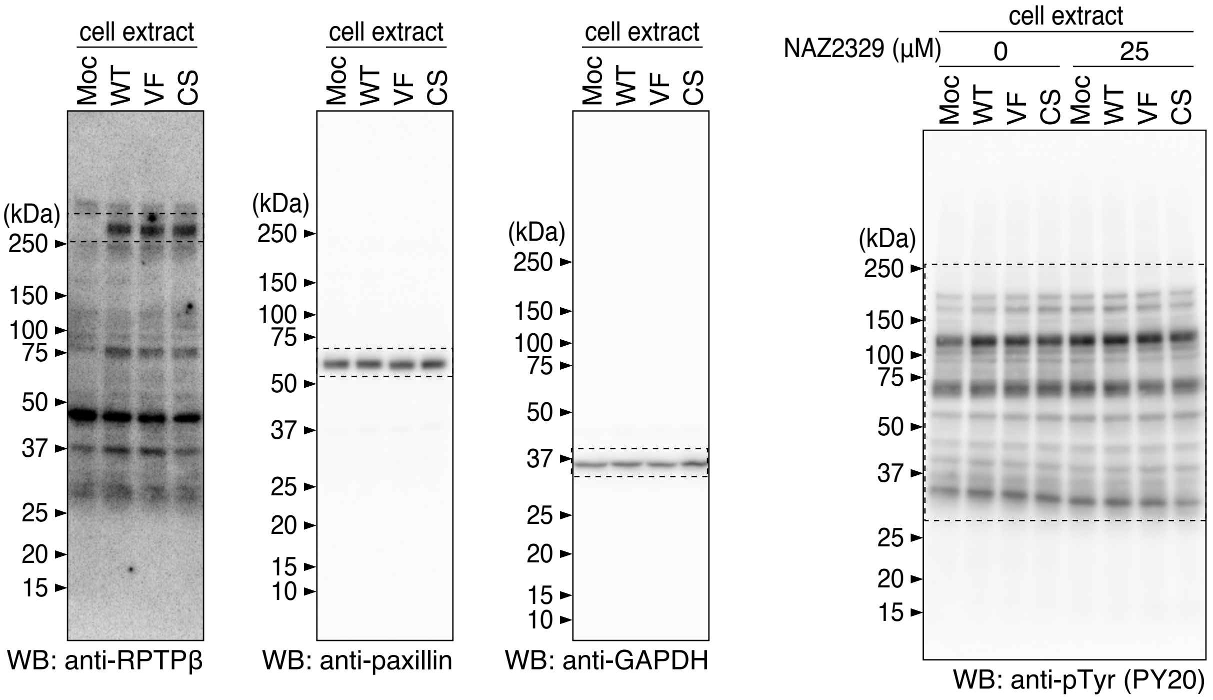

For Fig. 8B

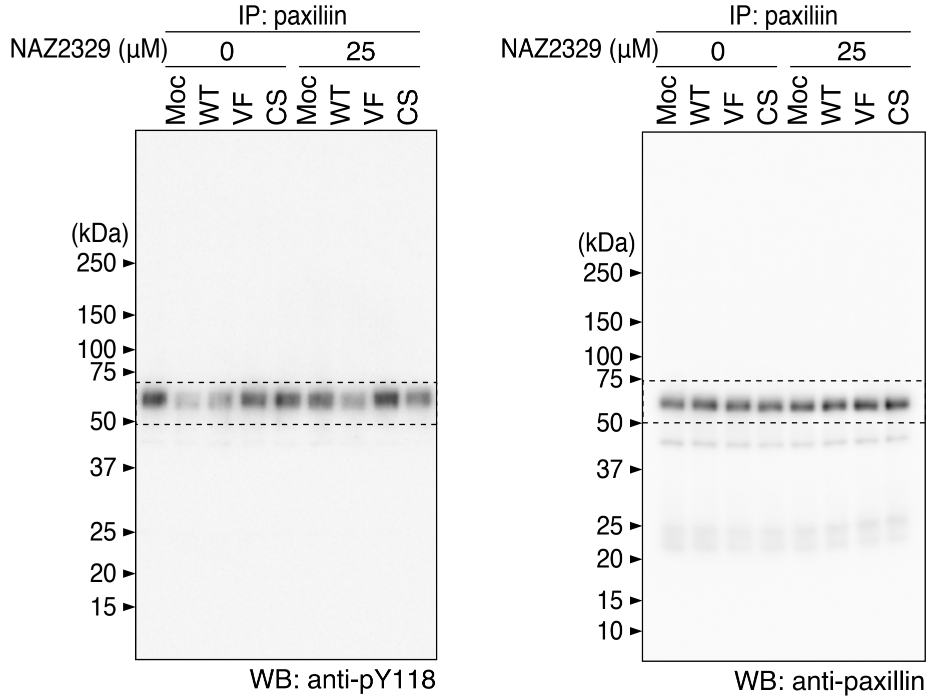

For Fig. 8D

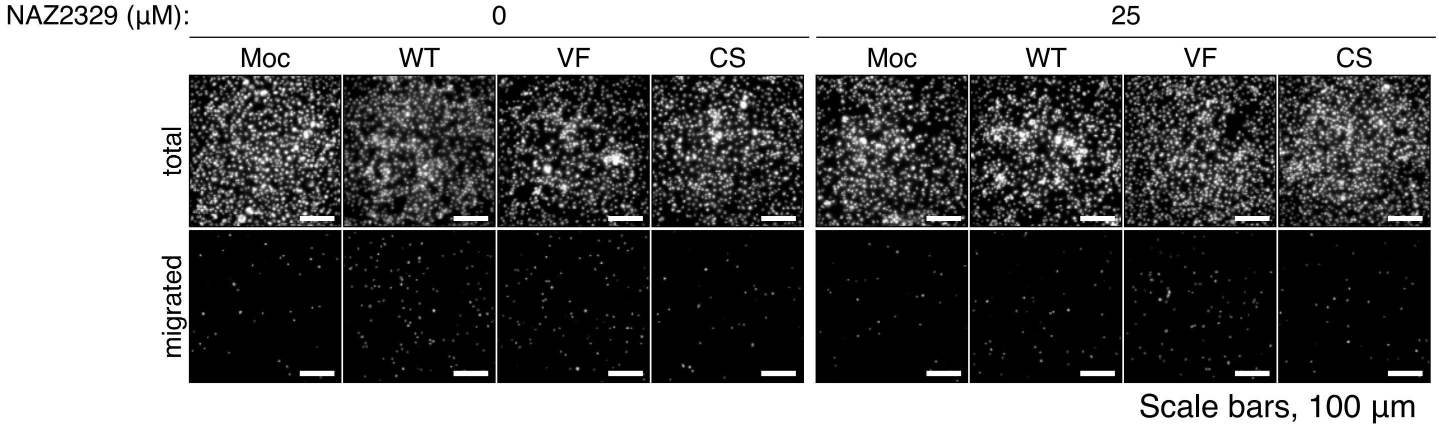

Supplementary Figure S8

Data collection and refinement statistics for the NAZ2329/PTPRZ1-D1 complex	
**Data Collection**	
Space group	$P2_12_12_1$
Cell Dimensions	
a,*b*,*c* (Å)	53.78, 72.31, 90.80
Resolution (Å)	2.53
Redundancy	4.5 (2.4)
Completeness (%)	87.2 (59.9)
I/sigma	22.8 (3.2)
R_{merge}	3.0 (22.3)
**Refinement**	
Resolution (Å)	2.53
No. of reflections	9992
R_{work} / R_{free}	21.0 /29.7
No. of atoms	
Protein	2276
Ligand/ion	32
Water	14
B-factors	
Protein	40.32
Ligand/ion	42.82
Water	31.02
R.m.s deviations	
Bond lengths (Å)	0.017
Bond angles (°)	1.894

Supplementary Table S1
